# Genome-Wide Association Study of Waterlogging Tolerance in Barley (*Hordeum vulgare* L.) Under Controlled Field Conditions

**DOI:** 10.3389/fpls.2021.711654

**Published:** 2021-08-26

**Authors:** Ana Borrego-Benjumea, Adam Carter, Min Zhu, James R. Tucker, Meixue Zhou, Ana Badea

**Affiliations:** ^1^Brandon Research and Development Centre, Agriculture and Agri-Food Canada, Brandon, MB, Canada; ^2^College of Agriculture, Yangzhou University, Yangzhou, China; ^3^Tasmanian Institute of Agriculture, University of Tasmania, Hobart, TAS, Australia

**Keywords:** barley, waterlogging tolerance, genome-wide associated study, marker-trait association, quantitative trait loci, candidate genes

## Abstract

Waterlogging is one of the main abiotic stresses severely reducing barley grain yield. Barley breeding programs focusing on waterlogging tolerance require an understanding of genetic loci and alleles in the current germplasm. In this study, 247 worldwide spring barley genotypes grown under controlled field conditions were genotyped with 35,926 SNPs with minor allele frequency (MAF) > 0.05. Significant phenotypic variation in each trait, including biomass, spikes per plant, grains per plant, kernel weight per plant, plant height and chlorophyll content, was observed. A genome-wide association study (GWAS) based on linkage disequilibrium (LD) for waterlogging tolerance was conducted. Population structure analysis divided the population into three subgroups. A mixed linkage model using both population structure and kinship matrix (Q+K) was performed. We identified 17 genomic regions containing 51 significant waterlogging-tolerance-associated markers for waterlogging tolerance response, accounting for 5.8–11.5% of the phenotypic variation, with a majority of them localized on chromosomes 1H, 2H, 4H, and 5H. Six novel QTL were identified and eight potential candidate genes mediating responses to abiotic stresses were located at QTL associated with waterlogging tolerance. To our awareness, this is the first GWAS for waterlogging tolerance in a worldwide barley collection under controlled field conditions. The marker-trait associations could be used in the marker-assisted selection of waterlogging tolerance and will facilitate barley breeding.

## Introduction

Waterlogging is a major abiotic stress that causes oxygen depletion and carbon oxide accumulation in the rhizosphere (Bailey-Serres and Voesenek, 2008) and has become one of the main concerns for crops limiting agricultural production globally. It is estimated that, worldwide, 10–16% of the arable soils are affected by waterlogging (Setter and Waters, 2003; Yaduvanshi et al., 2014). In western Canada, waterlogging has been identified as an important limiting factor for the crops grown, including barley. In the last decade, waterlogging was accountable for 52% of post-harvest claims for crop losses by farmers in Manitoba and Saskatchewan [Manitoba Agricultural Services Corporation (MASC), 2017; Saskatchewan Crop Insurance Corporation (SCIC), 2017]. Waterlogging occurs when there is excess moisture in the soil caused by high precipitation combined with poor soil drainage, resulting in anoxic and hypoxia within roots (Arduini et al., 2016). Waterlogging also causes an excess of ethylene and carbon dioxide that also increases metabolic toxins and microelements such as iron and manganese in soil solution or roots, reduces respiration, root conductivity to water, and nutrient uptake, thus affecting plant growth and survival (Setter and Waters, 2003).

Barley (*Hordeum vulgare* L.) is the fourth most important cereal crop globally and Canada's fourth-largest crop and is primarily used for livestock feed, malting, and food (FAOSTAT Production, 2020; Statistics Canada, 2020). Canada is the fourth largest barley producer and the second-largest malt exporter in the world. On average, each year, ~$1 billion is directly generated from the export of feed barley and malt [Canadian Agri-Food Trade Alliance (CAFTA), 2020]. Barley is more susceptible to waterlogging stress than other cereals (Setter and Waters, 2003). Waterlogging stress may cause significant yield losses in barley that vary from 10 to 50%, depending on factors such as the depth and duration of flooding, the development stage of the waterlogged plant, temperature (Setter et al., 1999) and type of soil (Pang et al., 2004). Waterlogging stress affects the genome-wide gene expression responses in barley roots, increasing the expression of many genes related to stress tolerance in barley roots, including glycolysis and fermentation-related genes, as well as ethylene-responsive element binding factors, and decreasing the expression of genes related to starch and sucrose metabolism, and nitrogen and amino acid metabolism (Borrego-Benjumea et al., 2020).

In barley, damages caused by soil waterlogging include chlorosis and premature leaf senescence, reduced root growth, tillering, dry matter accumulation, number and weight of kernels, and increased floral sterility (De San Celedonio et al., 2014, 2018; Masoni et al., 2016; Ploschuk et al., 2018; Sundgren et al., 2018). Under outdoor conditions in Argentina, Ploschuk et al. (2018) assessed tolerance to 14-days of early- or late-stage waterlogging of winter barley, which produced adventitious roots with 19% of aerenchyma. They showed that photosynthesis was reduced during waterlogging, but early-waterlogged plants were able to recover upon drainage with seed production reaching 85% of the controls, while late-waterlogged plants only attained 32% in seed production. Sayre et al. (1994) found that the growth stage of barley from leaf emergence to the booting stage is more sensitive to waterlogging, while Liu et al. (2020) reported that waterlogging close to heading is the most susceptible period, with yield losses primarily attributed to reductions in spikelet fertility and grain weight. In the Canadian Prairies, it has been projected increased precipitation in the coming years during May-June period (Blair et al., 2016). This is a critical period in the barley growing season in this region where increased precipitation reduces barley grain yield (Borrego-Benjumea et al., 2019). Therefore, it is important to develop cultivars tolerant to excess moisture and thus to increase the yield stability of barley.

Waterlogging tolerance is a complex quantitative trait under strong environmental influence with relatively low heritability of grain yield in barley (Hamachi et al., 1990). Due to this low heritability and dependency on environmental conditions, the direct selection of barley for waterlogging tolerance is time-consuming and less effective. Marker-assisted selection (MAS) is an effective approach that can improve the efficiency of breeding waterlogging-tolerant barley varieties and avoid environmental effects. MAS requires identifying appropriate quantitative trait loci (QTL) for traits associated with waterlogging tolerance, and the development of molecular markers closely linked to these traits. In barley, major QTL associated with waterlogging tolerance have revealed numerous genomic regions that affect important traits, such as chlorophyll fluorescence (Bertholdsson et al., 2015), root aerenchyma formation in cultivated and wild barley (Li et al., 2008; Zhang et al., 2016; Zhang X. et al., 2017), root membrane potential (Gill et al., 2017), root porosity (Broughton et al., 2015; Zhang et al., 2016), reactive oxygen species (ROS) formation (Gill et al., 2019), waterlogging score (Li et al., 2008; Zhou, 2011; Zhou et al., 2012), and yield components (Xue et al., 2010; Xu et al., 2012). All these major QTL have been mapped using doubled haploids (DH) populations from bi-parental crosses of contrasting phenotype parents for waterlogging. Although this approach has been the most applied and has been very successful in detecting many QTL for waterlogging tolerance in barley, few of the QTL reported have been successfully used in MAS.

Association mapping (AM) is another alternative to mapping QTL associated with complex traits in crops. The AM takes advantage of historic linkage disequilibrium to uncover genetic associations. Genome-wide association study (GWAS) requires high marker density because linkage disequilibrium (LD) is low in GWAS populations than in bi-parental populations. In GWAS, the mapping population consists of a diverse set of individuals or lines drawn from natural populations and breeding populations. GWAS has been used to detect QTL involved in response to waterlogging stress in various crops such as maize (Zhang et al., 2013), rice (Zhang M. et al., 2017), soybean (Cornelious et al., 2005) and wheat (Sundgren, 2018). In barley, GWAS has been used to identify QTL for not only agronomic traits, such as yield and yield components-related traits, using GWAS (Pasam et al., 2012; Locatelli et al., 2013; Tondelli et al., 2013; Pauli et al., 2014; Bellucci et al., 2017; Xu et al., 2018) but also tolerance to abiotic stresses such as salinity (Long et al., 2013; Fan et al., 2016; Mwando et al., 2020), drought (Varshney et al., 2012; Jabbari et al., 2018; Tarawneh et al., 2020), acid soil (Zhou et al., 2016), and low potassium (Ye et al., 2020) stress tolerance. However, no information is available for QTL mapping for waterlogging tolerance in barley by GWAS. In the present study, we assessed a worldwide barley collection for waterlogging stress tolerance under controlled field conditions. We evaluated the phenotypic and genetic diversity and the patterns of LD decay across the barley genome. We conducted GWAS for waterlogging tolerant traits, aiming to uncover novel genomic regions and identify marker-trait associations for waterlogging tolerance and confirm the previously identified genomic regions and single nucleotide polymorphism (SNP) marker associated with waterlogging tolerance. To our awareness, this is the first AM study for waterlogging stress tolerance in a worldwide barley collection under controlled field conditions.

## Materials and Methods

### Plant Material

A spring barley worldwide collection of 247 genotypes, including advanced breeding lines, cultivars, and landraces, was assembled and used in this study. The majority of genotypes were from Canada (30%), the USA (12%), China (10%), and Australia (8%). The rest were from 35 different countries.

### Field Experiment

The barley genotypes were evaluated for waterlogging tolerance in controlled field conditions in one location at the experimental station of the Brandon Research and Development Centre (49°52′ N, 99°58′ W) in two consecutive years (2016 and 2017). This location is a place where water is prone to accumulate, creating excess moisture problems. The soil has a sandy loam texture. The field trial area was leveled before seeding to ensure that all plants would be under the same water level. A ridge was built on the treatment side and was encircled by a plastic film to avoid water escape. The experimental design used was a randomized complete block design with three replications. Each plot represented one experimental unit, consisting of a single-row plot of 0.92 m length containing 25 seeds evenly distributed with 0.31 m spacing between rows. Seeds were sown in late May or early June following standard agronomic practices. Waterlogging-tolerant genotype Deder2 and waterlogging-sensitive genotype Franklin were used as checks. The waterlogging stress treatment was initiated at the tillering stage on the treatment side by adding the water to 0.5–1 cm above the soil surface. Waterlogging treatment was maintained at the same level and continued until the susceptible checks showed considerable stress symptoms (around 70% leaf symptom yellowing) and genotypic differences were easily distinguishable. The treatment duration was 9 and 7 days in 2016 and 2017, respectively. Then water in the waterlogged plots was drained out, and the plants were allowed to grow to maturity. Standard agronomic and cultural practices were applied to the other side of the field, used as control. The precipitation during the growing season was 394 and 245 mm in 2016 and 2017, respectively.

After full maturity, three individual plants were randomly harvested from each plot for analytical measurements. The traits evaluated included above-ground dry Biomass (BIO), number of spikes per plant (SP), number of grains per plant (GP), kernel weight per plant (KWP), plant height (PH), chlorophyll a+b content (CABC), chlorophyll carotenoids content (CCC), and waterlogging score (WLS) and were measured for 2 years in both treatment and control conditions. WLS was determined based on plant survival and leaf chlorosis (1 = not affected by waterlogging, 9 = plants died from waterlogging) (Supplementary Figure 1) after drainage (Zhou, 2011). For chlorophyll content determination, the pooled upper second leaf samples of six plants per plot under waterlogging conditions and three plants per plot under control were collected after the last day of treatment. From each pooled tissue leaf sample per plot, three biological replicates of 50 mg leaf tissue each were incubated with methanol. The absorbance, at wavelengths 470, 653, and 666 nm, was read using a spectrophotometer (SpectraMax 190 Microplate Reader). The number of pigments was calculated according to the formula from Lichtenthaler and Wellburn (1983). The mean values (three plants from each replicate × three replicates) of each plot sampled were subjected to statistical analysis.

### Statistical Analysis of Phenotypic Data

All data were analyzed using the statistical software JMP SAS version 14.1 (SAS Institute Inc., Cary, USA). The phenotypic data were analyzed using a mixed-effects model with genotype as a fixed effect, and year and replication nested within year as random effects. Least-squares means were estimated for waterlogging-treatment and control datasets within combined data across years. Pearson's correlation coefficient between pairs of traits was estimated to express the relationships between traits using the least-squares means across the combined years.

### Genotyping

The barley collection was grown in the greenhouse to generate plant tissue for DNA extraction using a standard potting mix, standard photoperiod conditions (16 h light), and 70% humidity. Genomic DNA from each genotype was extracted from pooled leaf tissue samples of four seedlings per genotype using a Qiagen DNeasy Plant Mini Kit (Qiagen GMbH, Germany). Before normalization, the quality and quantity of the extracted DNA were verified using a NanoDrop 1000 spectrophotometer (Thermo Scientific, Wilmington, Delaware, USA) and agarose gel electrophoresis, respectively. The samples were genotyped using the Barley 50K iSelect SNP Array (Illumina Inc., San Diego, CA, USA), containing 44,040 working assays (Bayer et al., 2017). All these data is presented in Supplementary Table 0. The SNP markers were further filtered using thresholds for minor allele frequency (MAF) of 0.05, missing rate of 0.20, and heterozygosity of 0.01. The final, filtered set of 35,926 SNPs was subsequently used for GWAS. Genotypes showing more than 0.02 heterozygous loci and call rates below 0.95 were also excluded from further analysis. There were 3551, 5798, 5486, 3904, 6497, 4233, and 5017 SNPs located at chromosomes 1 to 7, respectively, with 1,440 markers of unknown position.

### Population Structure, Kinship, and Linkage Disequilibrium Analyses

The population structure of the 247 barley genotypes, which represents the genetic similarity among genotypes, was assessed using the STRUCTURE program. Principal component analysis (PCA) (JMP Genomics 9.1) and neighbor-joining (NJ) (TASSEL 5.2.28) tree analysis were used as complementary approaches to confirm the results obtained using STRUCTURE. The STRUCTURE software version 2.3.4 (Pritchard et al., 2000) was used to estimate the most likely number of subpopulations (K) and the subpopulation coefficients (Q) by detecting allele frequency differences within the data and assigning individuals to those subpopulations based on analysis of likelihoods. A subset of 185 SNP markers, from the final filtered set of 35,926 SNP markers genotyped, were selected every ~25,000,000 bp on each chromosome through the barley genome, to ensure that the sample was representative. A Bayesian-based analysis was run using the admixture ancestry model with correlated allele frequencies (Falush et al., 2003). The burn-in period was set at 100,000, and the Markov Chain Monte Carlo (MCMC) repetitions at 100,000. The number of assumed clusters (k) was set from *k* = 1–7, and for each k, five runs were performed separately. The output data from STRUCTURE were assessed using STRUCTURE Harvester (Earl and von Holdt, 2012), where the optimum number of subpopulations (K) was determined by the Evanno method (Evanno et al., 2005). The K value was considered to be optimum, while ΔK reaches the maximum. Data for the most likely number of determining clusters (*K* = 3) were run to correctly align the clusters labeled from all five replications in STRUCTURE to obtain Q coefficients. The Q matrix with the lowest variance for the most likely number of k populations was selected and used as the fixed covariate in GWAS models. PCA was performed in JMP Genomics version 9.1 (SAS Institute Inc., Cary, USA). A K matrix representing the proportion of shared alleles for all pairwise comparisons in each population was computed. The neighbor-joining phylogenetic tree was implemented in TASSEL version 5.2.28 (Bradbury et al., 2007), which uses simple parsimony substitution models and is displayed by Archaeopteryx software.

The pairwise kinship values (kinship *K* matrix) for the association panel were calculated using the Identity-by-Descent (IBD) method in JMP Genomics 9.1. The *K* matrix estimates the relationships among the lines using marker data, rather than pedigree information, and computes the relationship measures directly while also accounting for selection and genetic drift. This kinship matrix was used for the subsequent GWAS in JMP Genomics as a random factor. The kinship coefficient was calculated and plotted vs. its frequency in the association panel.

Linkage disequilibrium (LD) analysis of the whole-genome and each of the seven chromosomes was performed in JMP Genomics 9.1 using 35,926 SNPs. Squared correlation coefficients (*r*^2^) were used to estimate the LD among the pairwise SNP markers using the maximum likelihood algorithm. To visualize the extent of LD, *r*^2^ was plotted against the map distance (bp), and a smoothing spline was fitted (λ = 100,000). The baseline *r*^2^ value was 0.1; an arbitrary value often used to describe LD decay (Zhu et al., 2008). The LD decay was estimated at the intersection point of the smoothing spline-fitting curve and the *r*^2^ value and was considered to estimate the extent of LD in the genome. All LD values above this critical *r*^2^ value were considered to be caused by genetic linkage.

### Genome-Wide Association Mapping Analysis and SNP Markers Identification

A total of 247 spring barley genotypes were used in this study based on genotypic and phenotypic data availability. Genome-wide association (GWA) mapping was conducted on each group using a total of 35,926 SNPs in JMP Genomics 9.1. Based on the population structural analysis, the general linear model (GLM) and mixed linear model (MLM) were run to investigate best-fit models in the current study to search for SNP associations with the traits. The MLM model considers both population structure (Q) and relative kinship (K) effects, and showed the best approximation of the expected cumulative distribution of *P*-values, and therefore, more effective in controlling false positives, and it was used for GWAS. The population structure matrix (*Q* matrix) evaluated using STRUCTURE and the kinship matrix analyzed using JMP Genomics 9.1 were used for the model. Association analysis was performed for each trait in each treatment for the phenotypic mean value of 2016 and 2017. The estimated effects for each allelic class were obtained directly from the mixed linear model. Adjusted *R*^2^ values were estimated from the linear regression model representing the percentage of phenotypic variation explained by the associated SNPs.

A GWAS threshold *P*-value of < 1.6 × 10^−4^ [−log_10_(*P*-value) < 3.8] was used for declaring significant-marker trait associations. They were based on the median of two threshold methods for determining significant *P*-values: a more stringent method of determining *P*-value (Wang et al., 2012), where the significance threshold is determined using the equation α = 1/m where m is the number of markers [–log_10_(*P*-value) < 4.5]; and a less stringent method (Chan et al., 2010) that is still widely accepted, where the bottom 0.1 percentile distribution of *P*-values is used as a threshold for significance [–log_10_ (*P*-value) < 3]. Manhattan plots were constructed with the chromosome position on the X-axis against –log(*P*-value) of all SNPs, and quantile-quantile (QQ) plots of observed *P*-values were constructed against expected *P*-values using JMP Genomics 9.1. The distribution of the QQ plot was considered to select the best model for each trait. The optimum model for each variable was determined as the one with the QQ plot with a smaller deviation from the normal distribution.

The GWAS was performed with the control, waterlogging treatment and relative datasets. The relative dataset was calculated as the relative difference between trait performance at the control and waterlogging treatment conditions. The markers that were significantly associated were assigned to QTL regions based on the trait, their chromosomal positions, and the estimated LD decay (1.460 Mbp). The identified QTL regions under control conditions were compared with QTL reported in previous studies in barley dealing with agronomic traits (Supplementary Table 1), and the waterlogging treatment and relative datasets were compared with QTL reported in previous studies in barley for waterlogging stress tolerance-related traits (Supplementary Table 2). When possible, BarleyMap (http://floresta.eead.csic.es/barleymap/find/) was used to collect cM positions from the POPSEQ_2017 genome map (Mascher et al., 2013) for significant markers in our study, to enable an approximate comparison between the physical and genetic map positions with the previous studies that reported QTL regions in genetic distance.

The phenotypic allele effect of each SNP locus, on the evaluated traits, was calculated through comparison of the average phenotypic value for each genotype for the specific allele with that of all genotypes (Mei et al., 2013).

### Candidate Gene Prediction

We opted to investigate the genes in the vicinity of each significant marker-trait associations, using a pre-defined flanking window of 200-kb upstream and downstream, below the 1.46 Mb LD decay detected in the current barley mapping collection (Lei et al., 2019). The identified genes were manually screened for potential annotations. Predicted genes were extracted from the barley reference genome assembly (IBSC v2; Mascher et al., 2017). Annotations were downloaded from Ensembl (http://plants.ensembl.org/Hordeum_vulgare/Info/Index) and AmiGO Gene Ontology (amigo.geneontology.org). The role of the potential candidate genes in response to abiotic stresses, especially waterlogging, was further examined using published literature.

## Results

### Phenotypic Data

Phenotypic variation was observed among genotypes for all traits in both control and waterlogging treatment (Table 1; Supplementary Figure 2). The frequency distribution of the genotypes for the investigated traits in the control and waterlogging treatment is presented in Supplementary Figure 3. In the control dataset, averaged over 2 years, BIO of the genotypes varied from 12.5 to 71.9 g, generated 5.4 to 22.4 SP, 9.2 to 312.2 GP, and weighted 0.2 to 14.5 g KWP. PH ranged from 18.5 to 95.8 cm, CABC varied from 0.89 to 1.54 mg/g leaf tissue, while CCC content varied from 0 to 0.17 mg/g leaf tissue (Table 1). After the exposure to waterlogging stress in the waterlogged dataset, averaged over 2 years, the genotypes varied in BIO from 1.7 to 36.3 g, generated 1.9 to 17.2 SP, 3.5 to 255.8 GP, and weighted 0.1 to 7.7 g KWP. PH ranged from 11.4 to 58.7 cm, CABC varied from 0.39 to 1.23 mg/g leaf tissue, while CCC varied from 0 to 0.12 mg/g leaf tissue (Table 1). As for WLS, the mean was 6.8, with a range from 4.7 to 8.8. Overall, for all genotypes, waterlogging stress reduced BIO, SP, GP, KWP, PH, CABC, and CCC by 72.1, 61.7, 67.5, 71.7, 45.1, 38.7, and 54.2%, respectively (Supplementary Figure 3). The coefficient of variation for the combined 2 years of data was higher for KWP (38.5 and 49.5% in control and waterlogging treatment, respectively), and lower for PH (16.0 and 17.4% in control and treatment conditions, respectively). There were highly significant (*P* < 0.05) genotypic differences both on individual and combined years for all traits except CABC and CCC (Table 1). The frequency distribution of all the traits generally fits a normal distribution (Supplementary Figure 3).

**Table 1:** Mean values and standard deviations of waterlogging-related traits observed under control and waterlogging treatment in field conditions for 247 spring barley genotypes.

**Trait**	**Year**	**Treatment**	**Mean**	**SD**	**Min**	**Max**	**Red.^a^**	**SE**	**CV (%)**	**G**
BIO (g)	2016	Control	37.3	16.9	2.5	86.7	71.5%	1.07	45.3	^***^
		Waterlogged	10.6	6.5	2.4	50.4		0.41	61.3	^***^
	2017	Control	42.8	7.7	20.3	79.0	72.6%	0.49	18.0	^***^
		Waterlogged	11.7	5.4	0.4	31.3		0.34	45.9	^***^
	2016/17	Control	40.1	11.1	12.5	71.9	72.1%	0.70	27.7	^***^
		Waterlogged	11.2	5.0	1.7	36.3		0.32	44.6	^***^
SP	2016	Control	12.2	4.9	2.0	25.0	75.1%	0.31	39.9	^***^
(number)		Waterlogged	3.0	2.4	0.0	24.2		0.15	78.7	^**^
	2017	Control	13.8	2.7	6.7	23.4	49.8%	0.17	19.8	^***^
		Waterlogged	6.9	1.8	2.2	12.2		0.11	25.8	^***^
	2016/17	Control	13.0	3.0	5.4	22.4	61.7%	0.19	23.3	^***^
		Waterlogged	5.0	1.6	1.9	17.2		0.10	32.3	^***^
GP	2016	Control	150.8	84.3	2.0	430.5	90.0%	5.35	55.9	^***^
(number)		Waterlogged	15.0	26.2	0.0	329.0		1.7	174.4	^***^
	2017	Control	167.5	49.0	13.4	362.0	47.1%	3.11	29.3	^***^
		Waterlogged	88.6	35.1	1.0	196.9		2.2	39.6	^***^
	2016/17	Control	159.2	57.0	9.2	312.2	67.5%	3.62	35.8	^***^
		Waterlogged	51.8	24.2	3.5	255.8		1.5	46.8	^***^
KWP (g)	2016	Control	6.3	3.7	0.0	19.0	92.5%	0.23	58.6	^***^
		Waterlogged	0.5	0.9	0.0	9.1		0.1	182.8	^***^
	2017	Control	6.6	2.2	0.3	16.1	51.9%	0.14	33.4	^***^
		Waterlogged	3.2	1.4	0.0	8.6		0.1	43.8	^***^
	2016/17	Control	6.4	2.5	0.2	14.5	71.7%	0.16	38.5	^***^
		Waterlogged	1.8	0.9	0.1	7.7		0.1	49.5	^***^
PH (cm)	2016	Control	73.5	13.7	17.5	101.3	54.8%	0.87	18.7	^***^
		Waterlogged	33.2	10.7	12.3	65.0		0.7	32.3	NS
	2017	Control	72.5	11.0	19.5	104.0	35.3%	0.70	15.2	^***^
		Waterlogged	46.9	8.6	7.8	70.5		0.5	18.4	^***^
	2016/17	Control	73.0	11.6	18.5	95.8	45.1%	0.74	16.0	^***^
		Waterlogged	40.1	7.0	11.4	58.7		0.4	17.4	^***^
CABC	2016	Control	1.13	0.2	0.66	1.55	41.3%	0.01	13.75	NS
(mg/g leaf tissue)		Waterlogged	0.66	0.3	0.03	1.39		0.02	38.12	NS
	2017	Control	1.39	0.1	0.96	1.67	36.6%	0.01	9.63	NS
		Waterlogged	0.88	0.2	0.39	1.47		0.01	21.79	^***^
	2016/17	Control	1.26	0.1	0.89	1.54	38.7%	0.01	8.41	NS
		Waterlogged	0.77	0.2	0.39	1.23		0.01	21.22	^**^
CCC	2016	Control	0.06	0.02	0.00	0.12	10.5%	0.00	42.75	NS
(mg/g leaf tissue)		Waterlogged	0.05	0.02	0.00	0.09		0.00	34.52	NS
	2017	Control	0.14	0.03	0.01	0.22	71.8%	0.00	24.02	NS
		Waterlogged	0.04	0.03	0.00	0.16		0.00	82.08	NS
	2016/17	Control	0.10	0.03	0.00	0.17	54.2%	0.00	33.38	NS
		Waterlogged	0.04	0.02	0.00	0.12		0.00	58.30	NS
WLS	2016	Waterlogged	6.9	1.2	3.3	9.0		0.08	17.5	^*^
(1–9 rating)	2017	Waterlogged	6.7	0.7	4.7	9.0		0.05	10.8	^***^
	2016/17	Waterlogged	6.8	0.8	4.7	8.8		0.05	12.0	^***^

*BIO, biomass; SP, spikes per plant; GP, grains per plant; KWP, kernel weight per plant; PH, plant height; CABC, chlorophyll a+b; CCC, carotenoids content; WLS, waterlogging score; SD, standard deviation; Red., Reduction; SE, standard error; CV, coefficient of variance; G, genotypic effect*.

a *Reduction ratio of all genotypes relative to control*.

* *Significant at P ≤ 0.05;*

** *significant at P ≤ 0.01;*

*** *significant at P ≤ 0.001; NS not significant*.

Correlations among traits under control and waterlogging treatment for 2016, 2017, and overall are shown in Table 2. In the combined 2 years of data, a negative correlation (*r* = −0.14 to −0.55; *P* ≤ 0.001) was observed between the WLS and all the traits (Table 2). Yield component traits (BIO, SP, GP, KWP, and PH) had high correlations in both control (*r* = 0.72 to 0.94; *P* ≤ 0.001) and waterlogging (*r* = 0.50–0.98; *P* ≤ 0.001) treatment.

**Table 2:** Pearson's phenotypic correlation coefficients among mean variables (least-squares entry means) of traits for control and waterlogging treatment measured in the spring barley collection in field conditions.

	**Year**	**Control**							
			**BIO**	**SP**	**GP**	**KWP**	**PH**	**CABC**	**CCC**
**Waterlogging treatment**	**2016**								
		BIO		0.83^***^	0.76^***^	0.70^***^	0.51^***^	−0.05 *NS*	0.07 *NS*
		SP	0.70^***^		0.87^***^	0.79^***^	0.44^***^	0.00 *NS*	0.07 *NS*
		GP	0.71^***^	0.79^***^		0.94^***^	0.48^***^	0.03 *NS*	0.04 *NS*
		KWP	0.71^***^	0.81^***^	0.96^***^		0.48^***^	0.02 *NS*	0.05 *NS*
		PH	0.25^***^	0.46^***^	0.28^***^	0.27^***^		−0.12^**^	0.15^***^
		CABC	0.23^***^	0.24^***^	0.12^**^	0.12^**^	0.50^**^		−0.55^***^
		CCC	0.18^***^	0.10^**^	0.08^*^	0.06 *NS*	0.29^***^	0.25^***^	
		WLS	−0.51^***^	−0.53^***^	−0.39^***^	−0.41^***^	−0.61^***^	−0.47^***^	−0.28^***^
	**2017**								
		BIO		0.71^***^	0.76^***^	0.80^***^	0.51^***^	−0.07 *NS*	0.10^**^
		SP	0.61^***^		0.73^***^	0.69^***^	0.22^***^	−0.01 *NS*	0.11^**^
		GP	0.61^***^	0.76^***^		0.96^***^	0.35^***^	−0.02 *NS*	0.04 *NS*
		KWP	0.55^***^	0.69^***^	0.96^***^		0.37^***^	−0.03 *NS*	0.07^*^
		PH	0.51^***^	0.32^***^	0.51^***^	0.49^***^		−0.03 *NS*	0.08^*^
		CABC	−0.22^***^	0.07 *NS*	0.12^**^	0.19^***^	−0.05 *NS*		0.02 *NS*
		CCC	0.05 *NS*	0.08^*^	0.05 *NS*	0.05 *NS*	0.04 *NS*	−0.11^**^	
		WLS	−0.52^***^	−0.31^***^	−0.32^***^	−0.27^***^	−0.42^***^	0.27^***^	−0.12^**^
	**2016/17**								
		BIO		0.79^***^	0.76^***^	0.72^***^	0.49^***^	0.02 *NS*	0.15^***^
		SP	0.61^***^		0.83^***^	0.76^***^	0.35^***^	0.06^*^	0.15^***^
		GP	0.53^***^	0.83^***^		0.94^***^	0.43^***^	0.05 *NS*	0.09^**^
		KWP	0.50^***^	0.80^***^	0.98^***^		0.44^***^	0.01 *NS*	0.06^*^
		PH	0.35^***^	0.55^***^	0.53^***^	0.52^***^		−0.10^***^	0.04 *NS*
		CABC	0.10^***^	0.30^***^	0.28^***^	0.30^***^	0.42^***^		0.18^***^
		CCC	0.08^**^	0.00 *NS*	−0.04 *NS*	−0.03 *NS*	0.06^*^	0.00 *NS*	
		WLS	−0.51^***^	−0.44^***^	−0.31^***^	−0.29^***^	−0.55^***^	−0.29^***^	−0.14^***^

*Control above diagonal, waterlogging treatment below diagonal. The correlations are estimated by the REML method*.

*BIO, biomass; SP, spikes per plant; GP, grains per plant; KWP, kernel weight per plant; PH, plant height; CABC, chlorophyll a+b; CCC, carotenoids content*.

* *Significant at P ≤ 0.05;*

** *significant at P ≤ 0.01;*

*** *significant at P ≤ 0.001; NS not significant*.

### Population Structure, Kinship, and Linkage Disequilibrium Analyses

**Figure 1:**
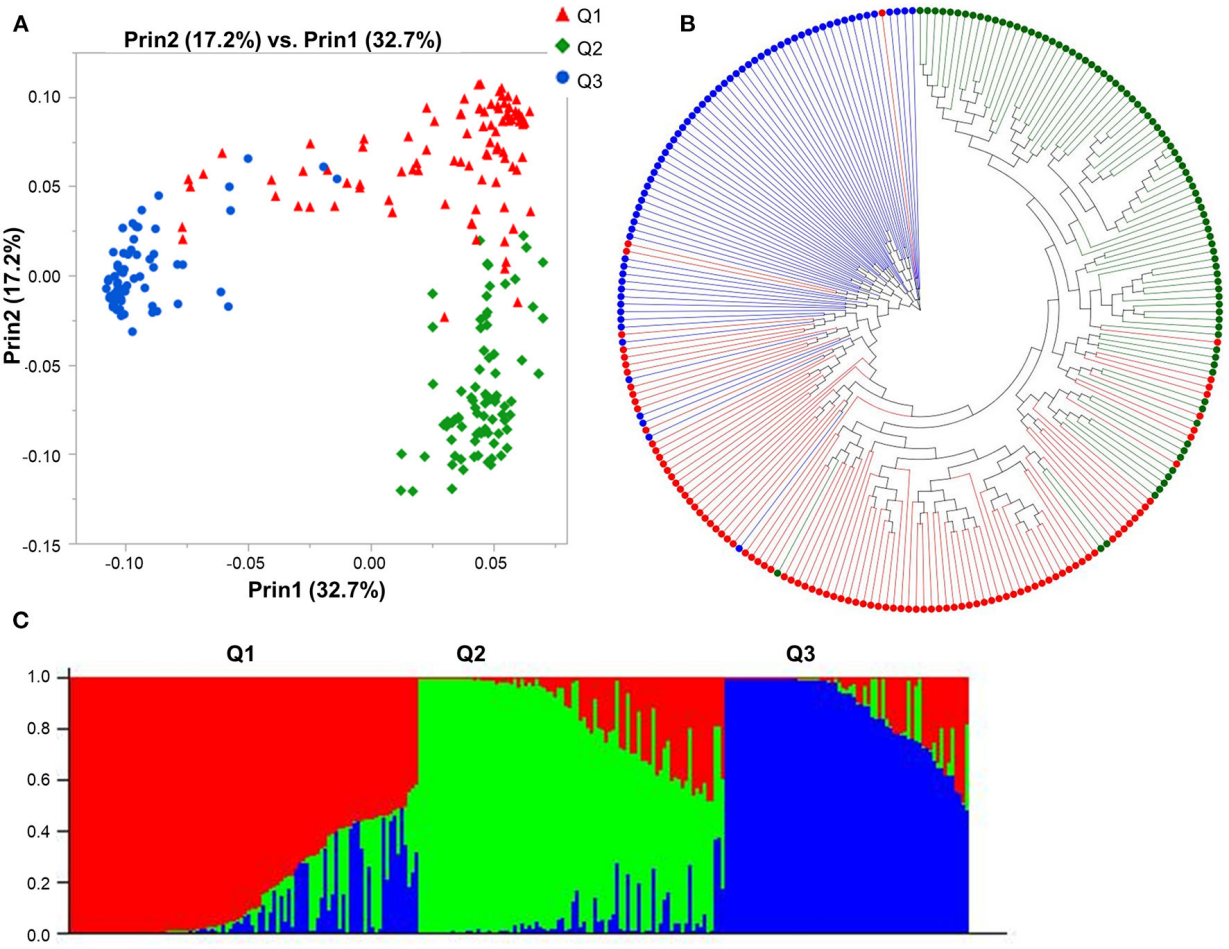
Model-based populations of spring barley collection: **(A)** Two-dimension distribution analyzed by principal component analysis (PCA) by JMP Genomics 9.1, **(B)** phylogenetic tree constructed by neighbor-joining (NJ) of genetic distance by TASSEL 5.2.28, and **(C)** Classification of three populations using STRUCTURE 2.3.4. The color code indicates the distribution of the accessions to different populations (Q1: red, Q2: green, Q3: blue) consistent in **(A–C)**.

The Bayesian approach implemented in STRUCTURE revealed the presence of three subpopulations with the highest likelihood for *K* = 3 (Supplementary Figure 4) and partitioned the 247 genotypes into three principal groups composed of 96, 83, and 68 genotypes each. Furthermore, the PCA analysis displayed consistent results, confirming the existence of the three subpopulations in agreement with the population structure analysis by STRUCTURE (Figure 1C), with the first two coordinates accounting for 72.5% of the genotypic variation (Figure 1A). The phylogenetic analysis partitioned the 247 genotypes into three principal groups, following the results obtained with STRUCTURE and PCA analyses (Figure 1B). Subpopulation 1 is mainly composed of genotypes from the USA (21), Canada (16), and Australia (8), subpopulation 2 included genotypes mainly from China (23), Australia (10), Switzerland (9), and Ethiopia (8), while subpopulation 3 included genotypes from Canada (55), US (9), Australia (1), Brazil (1) China (1), and Japan (1).

**Figure 2:**
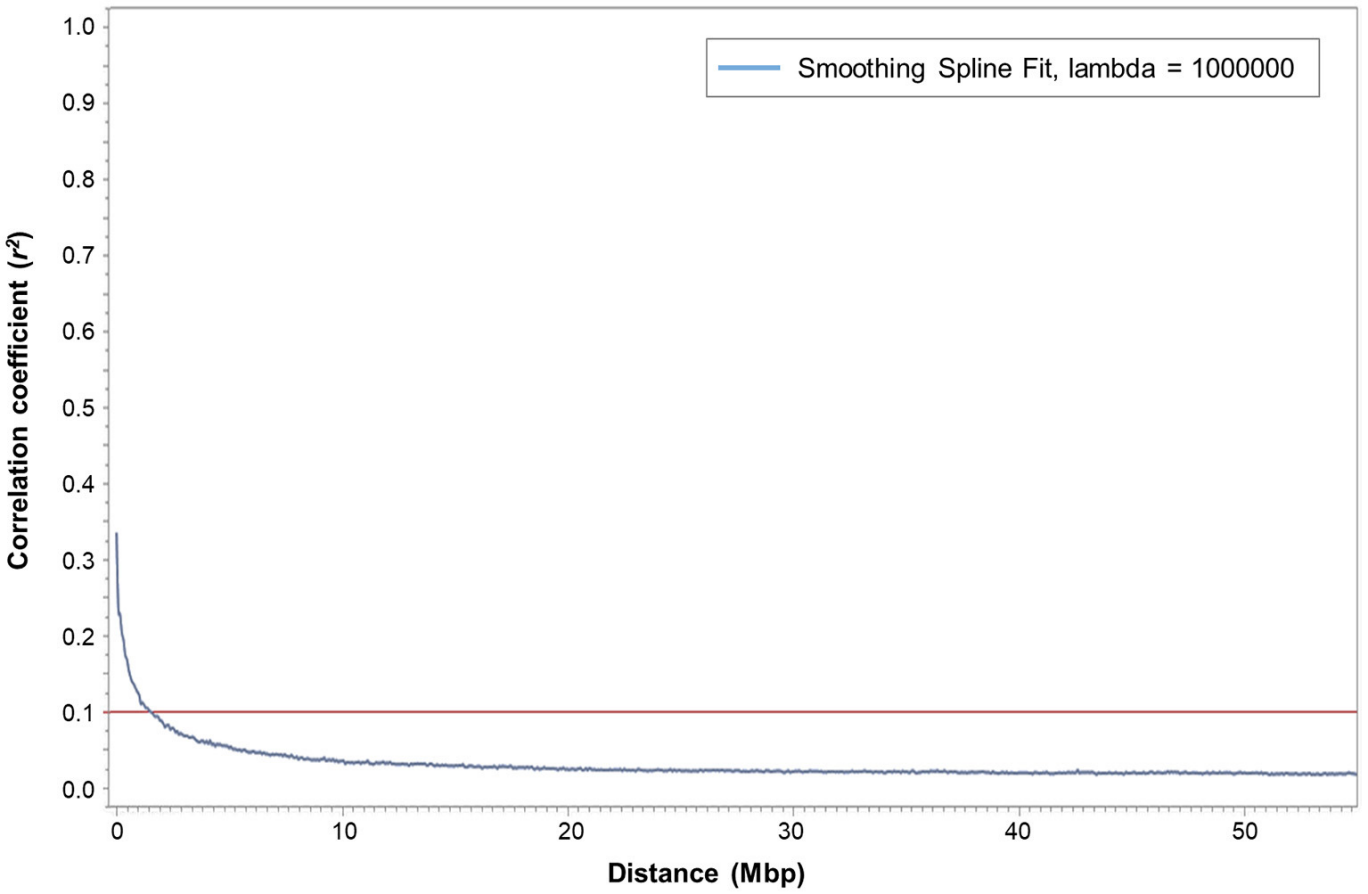
Plot of pairwise SNP linkage disequilibrium (LD) *r*^2^ value as a function of inter-marker genetic distances (Mbp) of 247 spring barley genotypes. The blue curve represents the smoothing spline regression model fit to LD decay. The red line represents the baseline *r*^2^ value at 0.1. The intersection of the fitted smoothing spline and *r*^2^ was observed at around 1,460,356 bp.

Squared correlation coefficient (*r*^2^) values among the marker pairs were used to estimate LD decay across all seven chromosomes (Figure 2) and each chromosome separately. The mean *r*^2^ ranged from 0.0178 (chromosome 5H) to 0.0261 (chromosome 4H). The arbitrary baseline *r*^2^ value was 0.1. The LD across all chromosomes decayed at 1,460,356 bp, whereas LD decay calculated for each chromosome separately ranged between 1,036,588 bp (chromosome 6H) and 2,290,772 bp (chromosome 1H). Based on the LD decay results, 35,926 SNPs (MAF > 0.05) will cover the entire barley genome and are adequate for GWAS with the assembled barley collection. Therefore, the mean window size of the QTL determined in this barley collection is ±1,460,356 bp from the highest peak of the significant marker-trait association.

### Association Mapping Analysis

**Figure 3:**
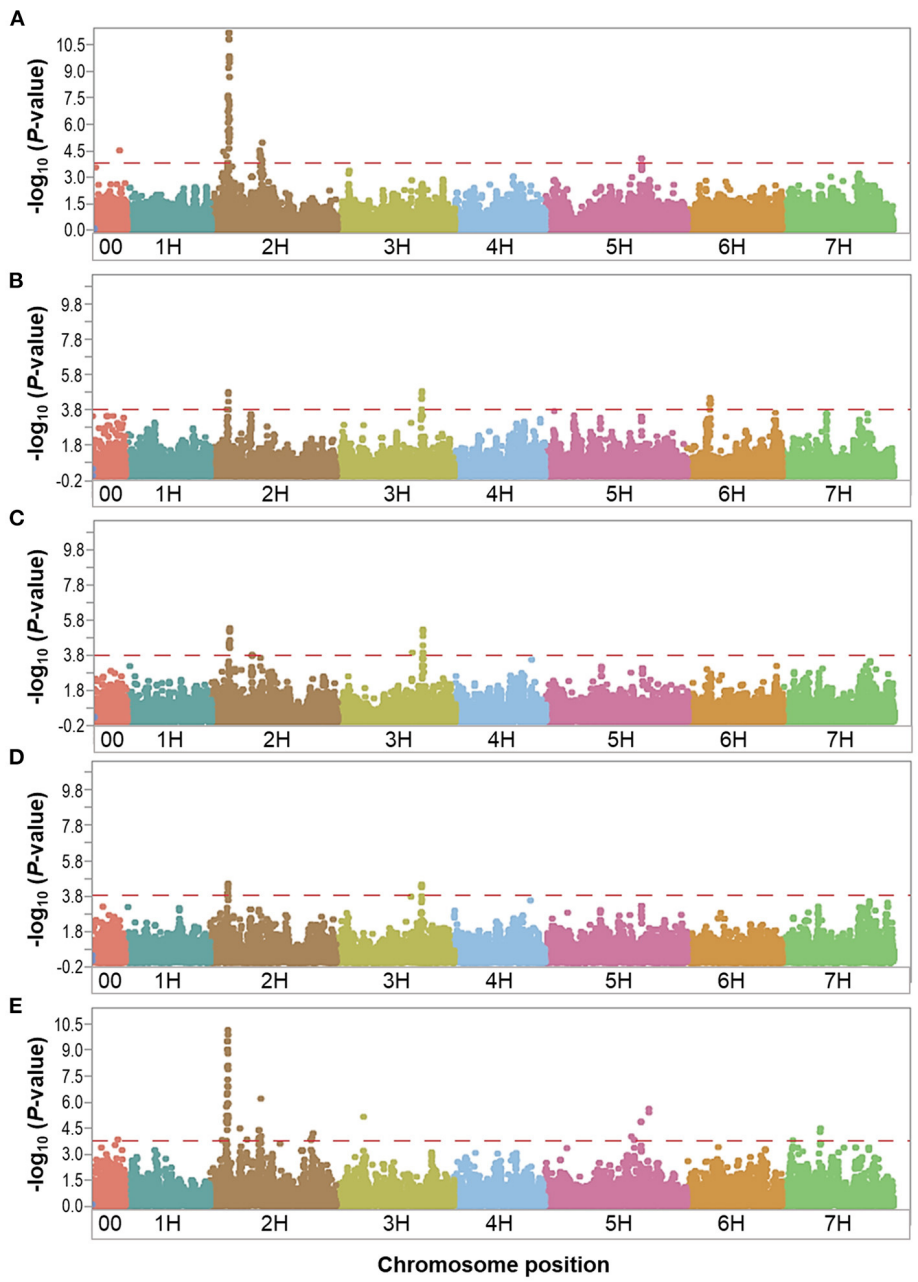
Manhattan plots resulting from the SNP-based GWAS in overall control under field conditions. Manhattan plots for Biomass (BIO), Spikes per plant (SP), Grains per plant (GP), Kernel weight per plant (KWP), and Plant height (PH) are shown in **(A–E)**, respectively, and the x-axis shows SNP loci along the seven barley chromosomes. The horizontal red line shows the genome-wide significance threshold *P*-value of 1.6 × 10^−4^ or –log_10_ (*P*-value) value of 3.8. GWAS was performed using the MLM (Q + K) model in JMP Genomics for the field traits.

**Figure 4:**
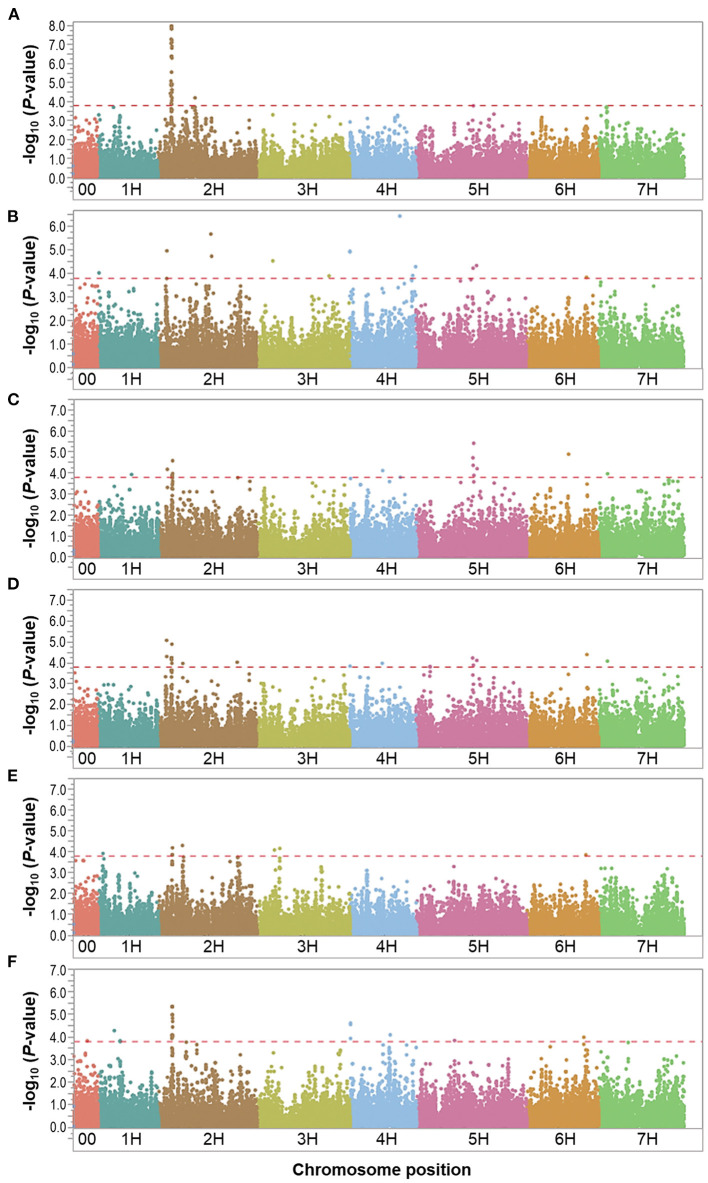
Manhattan plots resulting from the SNP-based GWAS in waterlogging treatment under field conditions. Manhattan plots for Biomass (BIO), Spikes per plant (SP), Grains per plant (GP), Kernel weight per plant (GWP), Plant height (PH), and Waterlogging score (WLS) are shown in **(A–F)**, respectively, and the x-axis shows SNP loci along the seven barley chromosomes. The horizontal red line shows the genome-wide significance threshold *P*-value of 1.6 × 10^−4^ or –log_10_ (*P*-value) value of 3.8. GWAS was performed using the MLM (Q + K) model in JMP Genomics for the field traits.

**Figure 5:**
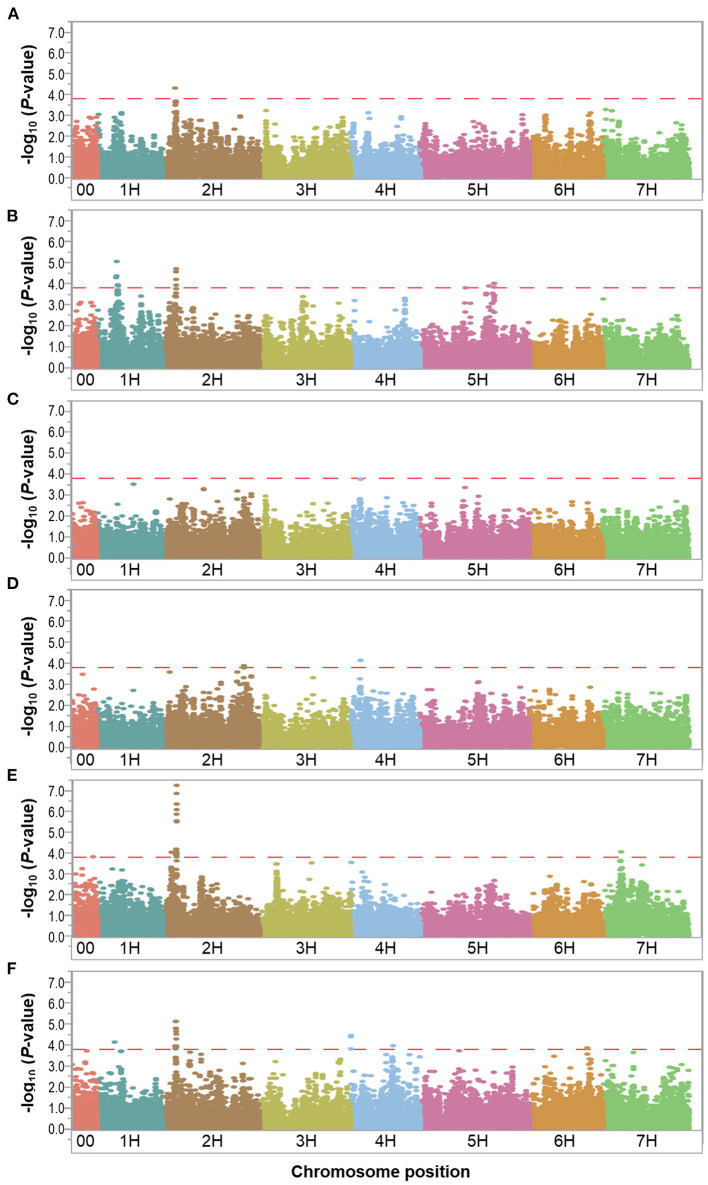
Manhattan plots resulting from the SNP-based GWAS identified in the relative dataset. Manhattan plots for Biomass (BIO), Spikes per plant (SP), Grains per plant (GP), Kernel weight per plant (GWP), Plant height (PH), and Waterlogging score (WLS) are shown in **(A–F)**, respectively, and the x-axis shows SNP loci along the seven barley chromosomes. The horizontal red line shows the genome-wide significance threshold *P*-value of 1.6 × 10^−4^ or –log_10_ (*P*-value) value of 3.8. GWAS was performed using the MLM (Q + K) model in JMP Genomics for the field traits.

We performed GWAS using 35,926 SNPs (with MAF > 0.05) for the control and waterlogging treatment conditions, as well as the relative difference between them using the phenotypic overall field experiment (mean value of 2016 and 2017), and a threshold *P*-value of < 1.6 × 10^−4^ [–log_10_(*P*-value) < 3.8]. Manhattan plots showed the significance of markers associated with the evaluated traits for the overall control, waterlogging treatment and relative datasets in Figures 3–**5**. QQ plots displayed that the expected and observed *P*-values initially matched, but eventually, they were delineated and deviated to indicate a reasonable positive (Supplementary Figures 5–7). Thus, the GWAS analysis is reliable and not likely to give false negatives (Figures 3–**5**).

#### Control Dataset

In the overall control conditions, the GWAS analysis identified a total of 92 markers significantly associated with BIO (52 markers), SP (18 markers), GP (23 markers), KWP (15 markers), and PH (62 markers), with some markers associated with multiple traits (Supplementary Table 3). Based on their position on chromosomes, these 92 significant markers mapped on 28 QTL regions on chromosomes 2H, 3H, 5H, 6H, and 7H, with each QTL region consisting of 1 to 34 markers, which included two regions for KWP; four regions for SP and GP; 12 regions for BIO; and 20 regions for PH (Figure 3; Supplementary Table 3). Some genomic regions were associated with multiple traits, indicating possible shared QTL between traits. For BIO in the control conditions, we found six genomic regions, out of 12, consisting of clusters of significant markers that mapped at 27.8, 29.1, 515.6, 542.4, and 547.4 Mbp on chromosome 2H, and at 600.9 Mbp on 5H (Table 3; Supplementary Table 3; Figure 3); each region consisted of clusters from 2 to up to 34 markers and explained on average from 6.2 to 12.3% of the phenotypic variation. Chromosome 2H consisted of the highest number of markers significantly associated with BIO (52 SNPs), of which BOPA2_12_30872 had the lowest *P*-value (6.3 × 10^−12^) with an allele effect size of 6.8 that individually explained 17.7% of phenotypic variation for BIO (Supplementary Table 3). The three genomic regions associated with SP in the control conditions were mapped at 29.7 Mbp on chromosome 2H, at 634.9 Mbp on chromosome 3H, and 35.4 Mbp on chromosome 6H and accounted on average for 5.8, 6.8, 6.9, and 6.4% of the phenotypic variation, respectively (Table 3; Supplementary Table 3). For GP in the control condition, we found two genomic regions at 29.7 Mbp (clusters of 14 SNPs) on chromosome 2H and 634.8 Mbp (7 SNPs) on 3H. On average, each genomic region explained between 6.9 and 7.1% of the phenotypic variation (Table 3; Supplementary Table 3). The two genomic regions associated with KWP in the control conditions were mapped at 29.7 Mbp on 2H (12 SNPs), and at 634.8 Mbp on 3H (3 SNPs). Each region explained, on average, from 6.1 to 6.7% of the phenotypic variation across the 2 years (Supplementary Table 3). For PH in the control conditions, we found nine genomic regions consisting of clusters of at least two significant markers that mapped at 28.5 Mbp (34 SNPs), 518.3 Mbp (2 SNPs), 523.4 Mbp (2 SNPs), 550.8 Mbp (2 SNPs), 723.7 Mbp (2 SNPs), and 727.6 Mbp (2 SNPs) on 2H, at 600.9 Mbp (2 SNPs), 613.3 Mbp on 5H (2 SNPs), and 75.1 Mbp on 7H (2 SNPs). Each region individually explained from 5.8 to 11.4% of the phenotypic variation (Supplementary Table 3).

**Table 3:** List of significant (*P* < 1.6 × 10^−4^) marker-trait associations detected by GWAS using the MLM (Q + K) model in JMP Genomics and favorable alleles (bold) for the assessed traits in the overall control conditions.

**Trait**	**Marker^a^**	**Ch**	**Physical position (bp)^b^**	**Genetic position (cM)^c^**	***P*** **-value**	***R*** **^2^ (%)^d^**	**MAF**	**Allele^e^**	**Additive effect**
BIO	JHI-Hv50k-2016-69385^f^^,^^*^	2H	19,064,497	13.31	3.60E-05	6.90	0.11	**T**/G	−6.16
	JHI-Hv50k-2016-71792^f^	2H	23,485,824	15.37	6.10E-05	6.40	0.19	**T**/C	−3.19
	JHI-Hv50k-2016-72991^*^	2H	27,836,916	18.91	3.40E-08	11.90	0.10	**A**/T	−5.85
	BOPA2_12_30872	2H	29,124,597	19.90	6.30E-12	17.70	0.18	**A**/G	−6.77
	JHI-Hv50k-2016-94875^f^^,^^*^	2H	496,673,313	55.01	3.00E-05	6.90	0.08	T/**C**	−5.23
	BOPA1_ABC08774-1-1-752^f^	2H	508,786,535		7.60E-05	6.30	0.05	A/**C**	−6.07
	JHI-Hv50k-2016-95073^f^	2H	515,576,575	58.64	4.40E-05	6.70	0.08	T/**C**	−4.79
	SCRI_RS_127347^f^	2H	519,110,344	58.64	5.80E-05	6.40	0.11	**T**/C	−4.56
	JHI-Hv50k-2016-97672^f^	2H	542,384,101	59.42	1.10E-04	6.00	0.06	A/**T**	−5.98
	JHI-Hv50k-2016-98186^f^	2H	547,420,281	59.42	1.10E-04	6.00	0.06	**C**/G	−5.98
	JHI-Hv50k-2016-336773^*^	5H	600,914,687	126.30	8.70E-05	6.20	0.07	**A**/T	−5.97
	JHI-Hv50k-2016-336814	5H	600,979,263		8.70E-05	6.20	0.07	T/**G**	−5.97
SP	JHI-Hv50k-2016-72991^*^	2H	27,836,916	18.91	1.50E-04	5.80	0.10	**A**/T	−1.25
	JHI-Hv50k-2016-73691^*^	2H	29,669,343		1.60E-05	7.60	0.15	A/**G**	−1.40
	JHI-Hv50k-2016-205634	3H	634,932,524	109.80	1.40E-05	7.50	0.35	**T**/C	1.09
	JHI-Hv50k-2016-382988	6H	35,396,724	43.77	3.40E-05	6.90	0.25	A/**G**	−0.96
GP	JHI-Hv50k-2016-73691^*^	2H	29,669,343		4.70E-06	8.40	0.15	A/**G**	−26.84
	JHI-Hv50k-2016-88492^f^	2H	134,404,110	55.01	1.50E-04	5.80	0.13	**A**/G	25.01
	JHI-Hv50k-2016-200577	3H	609,227,175	90.16	1.20E-04	6.00	0.27	**A**/G	15.27
	JHI-Hv50k-2016-205562^*^	3H	634,801,729	108.90	5.60E-06	8.20	0.44	**T**/C	17.77
KWP	JHI-Hv50k-2016-73691^*^	2H	29,669,343		3.30E-05	7.00	0.15	A/**G**	−1.06
	JHI-Hv50k-2016-205562^*^	3H	634,801,729	108.90	3.60E-05	6.80	0.44	**T**/C	0.70
PH	JHI-Hv50k-2016-69385^f^^,^^*^	2H	19,064,497	13.31	1.60E-04	5.80	0.11	**T**/G	−6.65
	JHI-Hv50k-2016-72991^*^	2H	27,836,916	18.91	1.80E-06	9.00	0.10	**A**/T	−5.90
	JHI-Hv50k-2016-73085^*^	2H	28,455,236	18.91	1.10E-05	7.80	0.41	**T**/C	9.61
	JHI-Hv50k-2016-80986^f^	2H	73,504,389	49.73	3.30E-05	6.90	0.07	T/G	−7.98
	JHI-Hv50k-2016-86347^f^	2H	112,364,666		1.40E-04	5.80	0.08	T/**C**	5.80
	JHI-Hv50k-2016-94875^f^^,^^*^	2H	496,673,313	55.01	1.40E-04	5.80	0.08	T/**C**	−5.81
	JHI-Hv50k-2016-95379^f^	2H	518,293,896	58.00	4.10E-05	6.70	0.08	A/**G**	−6.81
	JHI-Hv50k-2016-95777^f^	2H	523,378,213	58.64	1.20E-04	5.90	0.12	**A**/T	−6.05
	JHI-Hv50k-2016-98273^f^	2H	548,916,905		6.30E-07	9.70	0.06	T/**C**	−8.85
	JHI-Hv50k-2016-98501^f^	2H	550,839,094	59.35	9.00E-05	6.20	0.18	C/**G**	−4.88
	JHI-Hv50k-2016-127739	2H	723,652,876	122.90	1.50E-04	5.80	0.13	**T**/G	−4.62
	JHI-Hv50k-2016-129870	2H	727,578,152	125.20	6.20E-05	6.40	0.07	**A**/G	−7.42
	BOPA2_12_10532^f^	3H	67,560,907	45.82	7.00E-06	8.00	0.05	C/**G**	−7.71
	JHI-Hv50k-2016-330643	5H	587,449,015	114.70	9.60E-05	6.10	0.08	**T**/C	−5.60
	JHI-Hv50k-2016-332746	5H	591,637,968	120.10	1.50E-04	5.90	0.07	**A**/G	−7.35
	JHI-Hv50k-2016-336773^*^	5H	600,914,687	126.30	1.40E-05	7.50	0.07	**A**/T	−8.13
	BOPA2_12_31234^f^	5H	613,268,086	134.70	2.40E-06	8.80	0.07	A/**G**	−7.10
	JHI-Hv50k-2016-447227^f^	7H	11,309,509	7.78	1.60E-04	5.70	0.05	**A**/T	−6.84
	JHI-Hv50k-2016-468495^f^	7H	71,962,797	58.04	5.20E-05	6.60	0.10	A/**T**	−5.03
	JHI-Hv50k-2016-468869^f^	7H	75,059,390	59.80	3.30E-05	6.90	0.09	**A**/G	−5.21

*BIO, biomass; SP, spikes per plant; GP, grains per plant; KWP, kernel weight per plant; PH, plant height; Ch, chromosome number; MAF, minor allele frequency*.

a *The marker with the highest R^2^ in the genomic region is presented*.

b *Base pair positions of the marker in the chromosome based on a high-quality reference genome assembly for barley (Hordeum vulgare L.) (Mascher et al., 2017)*.

c *Genetic marker positions (cM) of the marker obtained from the POPSEQ_2017 genome map in BarleyMap (http://floresta.eead.csic.es/barleymap/find/) (Mascher et al., 2013)*.

d *R^2^ (%) indicates the percentage of phenotypic variation explained by the significant marker*.

e *Allele that is in bold text is the favorable allele for the trait assessed*.

f *Marker-trait associations that have different positions than the previously identified QTL for yield and yield-related traits published on barley under unstressed conditions*.

* *Putative QTL that may be associated with multiple traits*.

Under control conditions, six marker-trait associations representing genomic regions were associated with different traits (Table 3). On chromosome 2H, the marker JHI-Hv50k-2016-69385 at 19.0 Mbp was associated with the traits BIO and PH, with similar effects in phenotype (6.9 and 5.8% phenotypic variation, respectively); the marker JHI-Hv50k-2016-72991 at 27.8 Mbp was coincidental for BIO, SP, and PH, although with different effects in each trait (from 5.8 to 11.9% phenotypic variation); the marker JHI-Hv50k-2016-73691 located at 29.6 Mbp was associated with the traits SP, GP, and KWP; and the marker JHI-Hv50k-2016-94875 at 496.6 Mbp was shared by the traits BIO and PH (6.9 and 5.8% phenotypic variation, respectively). On chromosome 3H, the traits GP and KWP were associated with the same marker JHI-Hv50k-2016-205562 located at 634.8 Mbp, with 8.2 and 6.8% phenotypic variation, respectively (Table 3). Finally, on chromosome 5H, the traits BIO and PH were associated with the marker JHI-Hv50k-2016-336773 mapped at 600.9 Mbp with similar effects for the two traits (6.2 and 7.5% phenotypic variation, respectively).

#### Waterlogging Treatment Dataset

In the overall waterlogging treatment conditions, the GWAS analysis identified a total of 63 markers significantly associated with BIO (33 markers), SP (11 markers), GP (10 markers), KWP (20 markers), PH (4 markers), and WLS (25 markers), with some markers associated with multiple traits (Supplementary Table 4). Based on their position on chromosomes, these 63 significant SNPs were assigned to 24 QTL regions on chromosomes 1H, 2H, 3H, 4H, 5H, 6H, and 7H, with each region consisting of 1–30 markers, which included three regions for BIO; seven regions for GP; nine regions each for SP and KWP, four regions for PH, and five for WLS (Table 4; Figure 4). Some QTL regions were associated with multiple traits, indicating possible shared QTL between traits. For BIO in the waterlogging treatment conditions, three genomic regions were detected at 27.8, 28.3, and 516.6 Mbp on chromosome 2H. The genomic region at 28.3 Mbp consisted of the highest number of markers significantly associated with BIO (32 SNPs), explaining on average 9.5% of the phenotypic variation of the trait (Table 4; Figure 4). The most significant SNP marker, BOPA2_12_30872 had the lowest *P*-value (3.3 × 10^−8^) with an allele effect size of 2.8 that individually explained 11.8% of phenotypic variation for BIO (Supplementary Table 4). For SP in the waterlogging treatment conditions, we found two genomic regions consisting of clusters of two significant markers that mapped at 662.0 Mbp on 2H, and at 371,3 Mbp on 4H. Each region with an allele effect size of 1.2 individually explained from 7.4 to 8.57% of the phenotypic variation (Table 4). Clusters of two and three SNPs on chromosomes 2H at 29.6 Mbp and 5H at 568 Mbp, respectively, were significantly associated with GP in the waterlogging treatment conditions, which on average, accounted for 6.3 and 7.1% of phenotypic variation (Table 4; Supplementary Table 4). For KWP in the waterlogging treatment conditions, we found two genomic regions with at least two SNPs, at 16.8 Mbp (2 SNPs), and 29.7 Mbp on chromosome 2H (11 SNPs). On average, each genomic region explained between 6.1 and 6.9% of the phenotypic variation (Supplementary Table 4). The three genomic regions, with more than one SNP, associated with WLS in the waterlogging treatment conditions were found at 29.1 Mbp (17 SNPs) on chromosome 2H, and 0.37 and 569.8 Mbp (four and two SNPs, respectively) on 4H (Table 4; Supplementary Table 4; Figure 4); each region explained on average from 5.7 to 7.4% of the phenotypic variation. Chromosome 2H consisted of the highest number of markers significantly associated with WLS, of which BOPA2_12_30872 had the lowest *P*-value (7.5 × 10^−6^) with an allele effect size of 0.4 that individually explained 7.9% of phenotypic variation for WLS (Supplementary Table 4).

**Table 4:** List of significant (*P* < 1.6 × 10^−4^) marker-trait associations detected by GWAS using the MLM (Q + K) model in JMP Genomics and favorable alleles (bold) for assessed traits in the overall waterlogging treatment conditions.

**Trait**	**Marker^a^**	**Ch**	**Physical position (bp)^b^**	**Genetic position (cM)^c^**	***P*** **-value**	***R*** ^**2**^ **(%)^d^**	**MAF**	**Allele^e^**	**Additive effect**
BIO	JHI-Hv50k-2016-72991	2H	27,836,916	18.99	1.30E-05	7.6	0.10	**A**/T	−2.51
	BOPA2_12_30872^*^	2H	29,124,597	19.90	3.30E-08	11.8	0.18	**A**/G	−2.75
	JHI-Hv50k-2016-95223	2H	516,581,410	57.72	8.80E-05	6.2	0.10	**T**/C	−2.42
SP	JHI-Hv50k-2016-3532	1H	3,453,791	4.96	1.30E-04	6.2	0.07	A/**G**	0.85
	JHI-Hv50k-2016-68266 ^f^	2H	16,823,564	11.40	1.70E-05	7.4	0.08	**A**/G	1.34
	JHI-Hv50k-2016-109151	2H	662,018,769	82.51	3.70E-06	8.5	0.06	**A**/G	1.24
	JHI-Hv50k-2016-161633	3H	32,637,255	37.04	4.20E-05	6.7	0.06	A/**T**	0.99
	JHI-Hv50k-2016-225852^f^^,^^*^	4H	371,267	0.71	1.80E-05	7.3	0.06	T/**C**	1.17
	JHI-Hv50k-2016-262685	4H	607,200,114	85.84	7.40E-07	9.6	0.06	A/**G**	1.31
	JHI-Hv50k-2016-276624^f^	4H	645,759,577	117.30	7.20E-05	6.3	0.05	**T**/C	1.20
	JHI-Hv50k-2016-322832^*^	5H	569,308,558	97.51	8.20E-05	6.2	0.05	**A**/G	1.13
	BOPA2_12_11245^*^	5H	579,324,077		6.50E-05	6.4	0.06	C/**G**	1.07
GP	JHI-Hv50k-2016-68186^*^	2H	16,813,000	11.40	9.20E-05	6.1	0.11	**T**/C	10.47
	JHI-Hv50k-2016-73689^*^	2H	29,669,242		3.80E-05	6.8	0.14	A/**G**	−11.29
	JHI-Hv50k-2016-249670^f^^,^^*^	4H	512,990,076	54.32	1.10E-04	6.2	0.06	A/**G**	18.71
	JHI-Hv50k-2016-322832^*^	5H	569,308,558	97.51	6.50E-06	8.1	0.05	**A**/G	18.67
	BOPA2_12_11245^*^	5H	579,324,077		8.60E-05	6.2	0.06	C/**G**	15.07
	JHI-Hv50k-2016-410329^f^	6H	492,880,745	65.93	2.00E-05	7.3	0.07	**A**/C	18.63
	JHI-Hv50k-2016-449124^f^^,^^*^	7H	13,658,217	11.54	1.50E-04	5.8	0.35	**T**/C	7.27
KWP	JHI-Hv50k-2016-68186^f^^,^^*^	2H	16,813,000	11.40	1.30E-05	7.6	0.11	**T**/C	0.43
	JHI-Hv50k-2016-73689^*^	2H	29,669,242		2.00E-05	7.2	0.14	A/**G**	−0.44
	JHI-Hv50k-2016-82113	2H	79,456,923	49.73	1.40E-04	5.8	0.13	**T**/G	−0.34
	JHI-Hv50k-2016-127867	2H	724,202,574	120.80	1.30E-04	5.9	0.35	**A**/G	−0.26
	JHI-Hv50k-2016-249670^f^^,^^*^	4H	512,990,076	54.32	1.40E-04	6.0	0.06	A/**G**	0.68
	JHI-Hv50k-2016-322288	5H	568,058,046	97.51	8.10E-05	6.2	0.06	T/**G**	0.58
	BOPA2_12_11245^*^	5H	579,324,077		1.00E-04	6.0	0.06	C/**G**	0.55
	JHI-Hv50k-2016-424341^f^	6H	562,861,599	105.10	5.70E-05	6.5	0.06	**T**/G	0.56
	JHI-Hv50k-2016-449124^f^^,^^*^	7H	13,658,217	11.54	1.10E-04	6.0	0.35	**T**/C	0.27
PH	JHI-Hv50k-2016-73570	2H	29,307,953		9.00E-05	6.2	0.12	T/**C**	−3.30
	JHI-Hv50k-2016-80986	2H	73,504,389	49.73	7.00E-05	6.3	0.07	**T**/G	−5.31
	BOPA2_12_10968	3H	34,959,733	37.04	1.10E-04	6.0	0.06	**A**/G	−4.08
	JHI-Hv50k-2016-165725	3H	78,242,146		9.50E-05	6.2	0.30	**A**/G	3.61
WLS	JHI-Hv50k-2016-19217	1H	61,923,247		7.30E-05	6.3	0.07	T/**C**	−0.42
	BOPA2_12_30872^*^	2H	29,124,597	19.90	7.50E-06	7.9	0.18	**A**/G	0.39
	JHI-Hv50k-2016-225852^f^^,^^*^	4H	371,267	0.71	3.60E-05	6.8	0.06	T/**C**	−0.59
	BOPA1_3549-743^f^	4H	569,760,181	63.39	1.10E-04	6.0	0.40	**A**/G	0.26
	JHI-Hv50k-2016-421359^f^	6H	554,181,962	92.07	1.40E-04	5.9	0.08	A/**T**	−0.40

*BIO, biomass; SP, spikes per plant; GP, grains per plant; KWP, kernel weight per plant; PH, plant height; WLS, waterlogging score; Ch, chromosome number; MAF, minor allele frequency*.

a *The marker with the highest R^2^ in the genomic region is presented*.

b *Base pair positions of the marker in the chromosome based on a high-quality reference genome assembly for barley (Hordeum vulgare L.) (Mascher et al., 2017)*.

c *Genetic marker positions (cM) of the marker obtained from the POPSEQ_2017 genome map in BarleyMap (http://floresta.eead.csic.es/barleymap/find/) (Mascher et al., 2013)*.

d *R^2^ (%) indicates the percentage of phenotypic variation explained by the significant marker*.

e *Allele that is in bold text is the favorable allele for the trait assessed*.

f *Marker-trait associations that have different positions than the previously identified QTL for waterlogging stress-related traits published on barley under waterlogging conditions*.

* *Putative QTL that may be associated with multiple traits*.

Eight marker-trait associations associated with different traits were found in the waterlogging treatment conditions (Table 4). On chromosome 2H, the marker JHI-Hv50k-2016-68186 located at 16.8 Mbp was associated with the traits GP and KWP, although with different effects in each trait (from 6.1 to 7.6% phenotypic variation); the marker BOPA2_12_30872 located at 29.1 Mbp was coincidental for the traits BIO and WLS, with different effects on each trait (from 7.9 to 11.8% phenotypic variation); and the traits GP and KWP were associated to the same marker JHI-Hv50k-2016-73689 at 29.6 Mbp. On chromosome 4H, the traits SP and WLS were associated with the marker JHI-Hv50k-2016-225852 at 0.37 Mbp (7.3 and 6.8% phenotypic variation, respectively); and GP and KWP were associate to the same marker JHI-Hv50k-2016-249670 located at 512.9 Mbp (~6.1% phenotypic variation). On chromosome 5H, the traits SP and GP were associated with the marker JHI-Hv50k-2016-322832 regions at 569.3 Mbp; and the marker BOPA2_12_11245 at 579.3 Mbp was coincidental for the traits SP, GP, and KWP, with a similar effect for the three traits, ~6.2% phenotypic variation (Table 4). On chromosome 7H, the marker JHI-Hv50k-2016-449124 located at 13.6 Mbp was coincidental for the traits GP and KWP, with a similar effect.

Additionally, the analysis showed three markers on chromosome 2H co-localized in both control and waterlogging treatment conditions (Tables 3, 4). The marker JHI-Hv50k-2016-72991 located at 27.8 Mbp was found to be associated with BIO, SP, and PH under control, and with BIO under waterlogging treatment conditions; the marker BOPA2_12_30872 at 29.1 Mbp was identified in BIO under control, and BIO and WL under waterlogging treatment conditions; and the marker JHI-Hv50k-2016-80986 located at 73.5 Mbp was identified in PH under both control and waterlogging treatment conditions.

#### Relative Dataset

In order to find chromosomal regions that were significantly associated with waterlogging tolerance response, we analyzed the relative difference between the control and waterlogging treatment conditions. In the overall relative dataset, the GWAS analysis identified a total of 51 markers significantly associated with BIO (1 SNP), SP (17 SNPs), KWP (4 SNPs), PH (24 SNPs), and WLS (25 SNPs), with some markers associated with multiple traits (Supplementary Table 5). No significant markers were detected for GP in the relative dataset, unlike in the control and waterlogging treatment datasets. Based on their position on chromosomes, these 51 significant SNPs were assigned to 17 QTL regions on chromosomes 1H, 2H, 4H, 5H, 6H, and 7H, with each region consisting of 1 to 42 markers (Table 5; Figure 5; Supplementary Table 5). Some QTL regions were associated with multiple traits, indicating possible shared QTL between traits.

**Table 5:** List of significant (*P* < 1.6 × 10^−4^) marker-trait associations detected by GWAS using the MLM (Q + K) model in JMP Genomics and favorable alleles (bold) for assessed traits identified in the relative dataset.

**QTL**	**Trait**	**Marker^a^**	**Ch**	**Physical position (bp)^b^**	**Genetic position (cM)^c^**	***P*** **-value**	***R*** ^**2**^ **(%)^d^**	**MAF**	**Allele^e^**	**Additive effect**
QBIO.2H	BIO	JHI-Hv50k-2016-73118	2H	28,612,330	18.91	4.95E-05	6.64	0.43	A/**G**	6.17
QSP.1H-1	SP	JHI-Hv50k-2016-20766^f^	1H	107,293,686		5.17E-05	6.61	0.20	**T**/C	−6.33
QSP.1H-2		JHI-Hv50k-2016-20908	1H	187,645,763	47.94	4.31E-05	6.67	0.20	**T**/C	−6.38
QSP.1H-3		JHI-Hv50k-2016-21022	1H	241,516,420	47.94	8.79E-06	7.92	0.18	A/**G**	−6.71
QSP.1H-4		JHI-Hv50k-2016-22269	1H	296,548,971	47.94	1.15E-04	5.98	0.15	T/**G**	−6.12
QSP.1H-5		JHI-Hv50k-2016-22575	1H	303,086,870	47.94	1.15E-04	5.98	0.15	T/**C**	−6.12
QSP.2H		JHI-Hv50k-2016-73693^*^	2H	29,669,511		1.96E-05	7.24	0.06	**A**/C	13.31
QSP.5H-1		JHI-Hv50k-2016-312394^f^	5H	532,344,110		1.58E-04	5.79	0.08	T/**G**	10.59
QSP.5H-2		JHI-Hv50k-2016-332745	5H	591,637,898	120.07	1.30E-04	5.98	0.07	A/**G**	8.32
QSP.5H-3		JHI-Hv50k-2016-336773	5H	600,914,687	126.25	9.76E-05	6.07	0.07	A/**T**	8.15
QKWP.2H	KWP	JHI-Hv50k-2016-132004	2H	733,399,550	129.78	1.32E-04	5.85	0.06	T/**C**	6.98
QKWP.4H		JHI-Hv50k-2016-230103	4H	10,736,375	29.15	7.30E-05	6.31	0.06	A/**G**	9.95
QPH.2H-1	PH	BOPA2_12_30631^f^	2H	18,521,931	12.11	9.32E-05	6.10	0.50	**A**/G	2.91
QPH.2H-2		JHI-Hv50k-2016-73693^*^	2H	29,669,511		5.57E-08	11.46	0.06	A/**T**	12.99
QPH.7H		JHI-Hv50k-2016-457680	7H	32,776,909	29.96	8.89E-05	6.14	0.33	**A**/C	−4.13
QWLS.1H	WLS	JHI-Hv50k-2016-19217	1H	61,923,247	46.46	7.25E-05	6.29	0.07	T/**C**	−0.42
QWLS.2H		BOPA2_12_30872	2H	29,124,597	19.90	7.51E-06	7.94	0.18	**A**/G	0.39
QWLS.4H-1		JHI-Hv50k-2016-225850^f^	4H	370,915	0.71	4.05E-05	6.85	0.06	**T**/C	−0.58
QWLS.4H-2		BOPA1_3549-743^f^	4H	569,760,181	63.39	1.08E-04	5.99	0.39	**A**/G	0.26
QWLS.6H		JHI-Hv50k-2016-421359^f^	6H	554,181,962	92.07	1.36E-04	5.85	0.08	A/**T**	−0*s*.40

*BIO, biomass; SP, spikes per plant; KWP, kernel weight per plant; PH, plant height; WLS, waterlogging score; Ch, chromosome number; MAF, minor allele frequency*.

a *The marker with the highest R^2^ in the genomic region is presented*.

b *Base pair positions of the marker in the chromosome based on a high-quality reference genome assembly for barley (Hordeum vulgare L.) (Mascher et al., 2017)*.

c *Genetic marker positions (cM) of the marker obtained from the POPSEQ_2017 genome map in BarleyMap (http://floresta.eead.csic.es/barleymap/find/) (Mascher et al., 2013)*.

d *R^2^ (%) indicates the percentage of phenotypic variation explained by the significant marker*.

e *Allele that is in bold text is the favorable allele for the trait assessed*.

f *Marker-trait associations that have different positions than the previously identified QTL for waterlogging stress-related traits published on barley under waterlogging conditions*.

* *Putative QTL that may be associated with multiple traits*.

Since the focus of our study is waterlogging tolerance in barley, and the QTL found in the relative dataset are stable, we centered the discussion on these QTL which we named following the rule: “Q,” trait abbreviation, and chromosome number. One QTL associated with BIO, named QBIO.2H, was found on chromosome 2H and explained 6.6% of the phenotypic variation (Table 5; Figure 5; Supplementary Table 5; Supplementary Figure 8). This QTL also accounted for BIO under control and waterlogging treatment conditions (Tables 3, 4). Nine QTL for SP were detected on chromosomes 1H (QSP.1H-1, QSP.1H-2, QSP.1H-3, QSP.1H-4 and QSP.1H-5), 2H (QSP.2H), and 5H (QSP.5H-1, QSP.5H-2, QSP.5H-3), and explained 5.8–7.9% of the phenotypic variance (Table 5; Supplementary Table 5). Two QTL for KWP were detected on chromosomes 2H (QKWP.2H) and 4H (QKWP.4H) and explained 5.9–6.3% of the phenotypic variance (Table 5; Supplementary Table 5). For PH, three QTL were identified, located on chromosomes 2H (QPH.2H-1 and QPH.2H-2) and 7H (QPH.7H). The QTL accounted for 6.1–11.5% of the phenotypic variance (Table 5; Supplementary Table 5). The QTL QWT.PH.2H-2 also accounted for PH under control and waterlogging treatment conditions (Tables 3, 4). Five QTL affecting WLS were identified and they accounted for 5.9–7.9% of the phenotypic variance (Table 5; Supplementary Table 5). They were located in chromosomes 1H (QWLS.1H), 2H (QWLS.2H), 4H (QWLS.4H-1 and QWLS.4H-2) and 6H (QWLS.6H). These five QTL also accounted for WLS under waterlogging treatment (Table 4).

One genomic region was associated with various traits in the relative dataset (Table 5). On chromosome 2H, QTL QWT.BIO.2H, QWT.SP.2H and QWT.PH.2H-2 located at 28-29 Mbp were associated with BIO, SP, and PH, respectively, although with different effects in each trait (6.6–11.5% of phenotypic variation).

### Candidate Genes

A total of 205, 190, and 156 genes were located within a 200-kb genomic region up- and down-stream centered from 32, 26 and 18 significant marker-trait associations in control (Supplementary Table 6), waterlogging treatment conditions (Supplementary Table 7) and relative dataset (Supplementary Table 8), respectively. Among those markers, 22, 19, and 14, from control, waterlogging treatment and relative datasets, respectively, were located inside genes. We focused on these genes and identified nine possible candidate genes associated with the measured traits under the control (Table 6), 13 possible candidate genes associated with these traits under the waterlogging treatment conditions (Table 7), and eight possible candidate genes associated with the measured traits in the relative dataset (Table 8).

**Table 6:** Summary of potential candidate genes that contain significant markers associated with the assessed traits under control conditions.

**Marker**	**Trait**	**Ch**	**Marker position (bp)**	**Gene ID**	**Start (bp)**	**End (bp)**	**Gene description**
JHI-Hv50k-2016-71792	BIO	2H	23,485,824	HORVU2Hr1G011650	23,481,402	23,486,230	Undescribed protein
BOPA2_12_30872	BIO	2H	29,124,597	HORVU2Hr1G013400	29,123,724	29,127,894	Pseudo-response regulator 7
BOPA1_ABC08774-1-1-752	BIO	2H	508,786,535	HORVU2Hr1G071330	508,785,994	508,794,465	Glycine–tRNA ligase
JHI-Hv50k-2016-95073	BIO	2H	515,576,575	HORVU2Hr1G071980	515,568,391	515,580,748	Heparan-α-glucosaminide N-acetyltransferase
SCRI_RS_127347	BIO	2H	519,110,344	HORVU2Hr1G072400	519,108,149	519,110,415	Cytochrome P450 superfamily protein
JHI-Hv50k-2016-98186	BIO	2H	547,420,281	HORVU2Hr1G075950	547,420,245	547,422,120	Zinc finger homeodomain 1
JHI-Hv50k-2016-336773	BIO, KWP	5H	600,914,687	HORVU5Hr1G096320	600,914,511	600,916,443	UDP-Glycosyltransferase superfamily protein
JHI-Hv50k-2016-94875	BIO, PH	2H	496,673,313	HORVU2Hr1G070320	496,671,113	496,676,443	Yellow stripe like 6
JHI-Hv50k-2016-88492	GP	2H	134,404,110	HORVU2Hr1G033730	134,403,521	134,420,781	Proteasome maturation factor UMP1 family protein
JHI-Hv50k-2016-205562	GP, KWP	3H	634,801,729	HORVU3Hr1G091170	634,799,742	634,804,670	Receptor kinase 2
JHI-Hv50k-2016-73085	PH	2H	28,455,236	HORVU2Hr1G013020	28,452,211	28,456,166	Trichome birefringence-like 4
JHI-Hv50k-2016-86347	PH	2H	112,364,666	HORVU2Hr1G030520	112,360,955	112,366,308	Protein kinase superfamily protein
JHI-Hv50k-2016-95777	PH	2H	523,378,213	HORVU2Hr1G072750	523,377,399	523,379,178	Protein Terminal flower 1
JHI-Hv50k-2016-98501	PH	2H	550,839,094	HORVU2Hr1G076520	550,832,263	550,840,111	Pectinesterase family protein
JHI-Hv50k-2016-127739	PH	2H	723,652,876	HORVU2Hr1G111640	723,652,502	723,658,875	Plasma membrane ATPase
JHI-Hv50k-2016-129870	PH	2H	727,578,152	HORVU2Hr1G113190	727,572,166	727,583,311	Alpha-N-acetylglucosaminidase
BOPA2_12_10532	PH	3H	67,560,907	HORVU3Hr1G021150	67,560,410	67,562,131	Gigantea protein (GI)
JHI-Hv50k-2016-332746	PH	5H	591,637,968	HORVU5Hr1G093390	591,633,650	591,639,220	Solute carrier family 22 member 1
BOPA2_12_31234	PH	5H	613,268,086	HORVU5Hr1G101820	613,267,130	613,268,378	Undescribed protein
JHI-Hv50k-2016-447227	PH	7H	11,309,509	HORVU7Hr1G008690	11,307,419	11,313,973	Protein kinase superfamily protein
JHI-Hv50k-2016-468495	PH	7H	71,962,797	HORVU7Hr1G034400	71,959,645	71,963,636	Unknown function
JHI-Hv50k-2016-468869	PH	7H	75,059,390	HORVU7Hr1G034990	75,057,969	75,067,902	Kinesin-related protein 11

*BIO, biomass; SP, spikes per plant; GP, grains per plant; KWP, kernel weight per plant; PH, plant height; WLS, waterlogging score; Ch, chromosome number*.

**Table 7:** Summary of potential candidate genes that contain significant markers associated with the assessed traits under waterlogging treatment conditions.

**Marker**	**Trait**	**Ch**	**Marker position (bp)**	**Gene ID**	**Start (bp)**	**End (bp)**	**Gene description**
JHI-Hv50k-2016-95223	BIO	2H	516,581,410	HORVU2Hr1G072140	516,578,216	516,583,796	Uridylate kinase
BOPA2_12_30872	BIO, WLS	2H	29,124,597	HORVU2Hr1G013400	29,123,724	29,127,894	Pseudo-response regulator 7
JHI-Hv50k-2016-161633	SP	3H	32,637,255	HORVU3Hr1G014290	32,636,782	32,639,178	Delta(8)-Delta(7) sterol isomerase
JHI-Hv50k-2016-276624	SP	4H	645,759,577	HORVU4Hr1G090640	645,757,976	645,762,395	E3 ubiquitin-protein ligase RFWD3
JHI-Hv50k-2016-109151	SP	2H	662,018,769	HORVU2Hr1G094030	662,015,232	662,019,114	Ubiquitin-conjugating enzyme 3
JHI-Hv50k-2016-3532	SP	1H	3,453,791	HORVU1Hr1G001480	3,453,090	3,454,077	Undescribed protein
JHI-Hv50k-2016-322832	SP, GP	5H	569,308,558	HORVU5Hr1G083110	569,293,089	569,309,305	Leucine-rich repeat receptor-like protein kinase
BOPA2_12_11245	SP, GP, KWP	5H	579,324,077	HORVU5Hr1G087730	579,322,710	579,324,607	13S globulin seed storage protein 2
JHI-Hv50k-2016-225852	SP, WLS	4H	371,267	HORVU4Hr1G000090	369,520	374,029	RING/U-box superfamily protein
JHI-Hv50k-2016-410329	GP	6H	492,880,745	HORVU6Hr1G070750	492,878,969	492,884,688	E3 ubiquitin-protein ligase makorin
JHI-Hv50k-2016-249670	GP, KWP	4H	512,990,076	HORVU4Hr1G061070	512,989,821	512,992,961	C2H2-like zinc finger protein
JHI-Hv50k-2016-82113	KWP	2H	79,456,923	HORVU2Hr1G025510	79,452,094	79,457,099	B3 domain-containing protein
JHI-Hv50k-2016-424341	KWP	6H	562,861,599	HORVU6Hr1G087000	562,860,368	562,867,337	Heparanase-like protein 3
JHI-Hv50k-2016-127867	KWP	2H	724,202,574	HORVU2Hr1G111780	724,201,388	724,204,020	Receptor-like protein kinase 4
JHI-Hv50k-2016-322288	KWP	5H	568,058,046	HORVU5Hr1G082670	568,057,965	568,060,772	Undescribed protein
BOPA2_12_10968	PH	3H	34,959,733	HORVU3Hr1G015050	34,956,640	34,962,056	Enolase-phosphatase E1
JHI-Hv50k-2016-165725	PH	3H	78,242,146	HORVU3Hr1G022270	78,241,796	782,431,36	Pentatricopeptide repeat 336
BOPA1_3549-743	WLS	4H	569,760,181	HORVU4Hr1G069280	569,757,996	569,767,162	Alpha-L-fucosidase 2
JHI-Hv50k-2016-19217	WLS	1H	61,923,247	HORVU1Hr1G017900	61,919,204	61,923,605	Transcription factor PIF3

*BIO, biomass; SP, spikes per plant; GP, grains per plant; KWP, kernel weight per plant; PH, plant height; WLS, waterlogging score; Ch, chromosome number*.

**Table 8:** Summary of potential candidate genes that contain significant markers associated with the assessed traits identified in the relative dataset.

**Marker**	**Trait**	**Ch**	**Marker position (bp)**	**Gene ID**	**Start (bp)**	**End (bp)**	**Gene description**
JHI-Hv50k-2016-20766	SP	1H	107,293,686	HORVU1Hr1G024060	107,289,291	107,295,231	Arginine/serine-rich splicing factor 35
JHI-Hv50k-2016-20908	SP	1H	187,645,763	HORVU1Hr1G031370	187,632,592	187,656,006	tRNA pseudouridine synthase A1
JHI-Hv50k-2016-21022	SP	1H	241,516,420	HORVU1Hr1G036060	241,482,945	241,524,027	Cationic amino acid transporter 2
JHI-Hv50k-2016-22269	SP	1H	296,548,971	HORVU1Hr1G041530	296,548,421	296,553,191	Predicted protein
JHI-Hv50k-2016-22575	SP	1H	303,086,870	HORVU1Hr1G041960	303,085,891	303,088,081	Unknown function
JHI-Hv50k-2016-312394	SP	5H	532,344,110	HORVU5Hr1G071230	532,343,847	532,345,355	Unknown function
JHI-Hv50k-2016-332745	SP	5H	591,637,898	HORVU5Hr1G093390	591,633,650	591,639,220	Solute carrier family 22 member 1
JHI-Hv50k-2016-336773	SP	5H	600,914,687	HORVU5Hr1G096320	600,914,511	600,916,443	UDP-Glycosyltransferase superfamily protein
JHI-Hv50k-2016-132004	KWP	2H	733,399,550	HORVU2Hr1G114940	733,394,545	733,400,877	Cyclic nucleotide gated channel 8
JHI-Hv50k-2016-457680	PH	7H	32,776,909	HORVU7Hr1G022410	32,775,788	32,780,170	RNA-binding protein mde7
JHI-Hv50k-2016-19217	WLS	1H	61,923,247	HORVU1Hr1G017900	61,919,204	61,923,605	Transcription factor PIF3
BOPA2_12_30872	WLS	2H	29,124,597	HORVU2Hr1G013400	29,123,724	29,127,894	Pseudo-response regulator 7
JHI-Hv50k-2016-225850	WLS	4H	370,915	HORVU4Hr1G000090	369,520	374,029	RING/U-box superfamily protein
BOPA1_3549-743	WLS	4H	569,760,181	HORVU4Hr1G069280	569,757,996	569,767,162	Alpha-L-fucosidase 2

*SP, spikes per plant; KWP, kernel weight per plant; PH, plant height; WLS, waterlogging score; Ch, chromosome number*.

Significant markers associated with BIO in control conditions were inside genes (HORVU2Hr1G013400, HORVU2Hr1G071330, HORVU2Hr1G072400, HORVU2Hr1G075950, HORVU5Hr1G096320, and HORVU2Hr1G070320) involved in the regulation of the circadian clock, regulation of flowering time and development, embryogenesis, grain size and development, plant growth, development and senescence (Table 6). The role of the genes HORVU5Hr1G096320 and HORVU2Hr1G033730 harboring the markers JHI-Hv50k-2016-336773 and JHI-Hv50k-2016-88492, respectively, associated with GP and KWP traits were known to be essential in the regulation of seed development and grain size (Table 6). Several genes (HORVU2Hr1G013020, HORVU2Hr1G076520, and HORVU7Hr1G034990) associated with the significant markers for PH trait were known to be involved in cell wall processes, such as synthesis and deposition of secondary wall cellulose, modulation of cell wall mechanical stability during fruit ripening, cell wall extension during pollen germination and pollen tube growth, abscission, stem elongation, tuber yield and root development, microtubule-binding proteins involved in the microtubule control of cellulose microfibril order and cell wall strength. Some other genes (HORVU2Hr1G030520, HORVU5Hr1G093390, and HORVU7Hr1G008690) play a role in cell cycle regulation processes, such as modulating vesicle transport and channel activities, and specific transport of various substrates. Another group of genes (HORVU2Hr1G072750, HORVU2Hr1G111640, HORVU2Hr1G113190, and HORVU3Hr1G021150) regulate plant growth and reproductive development, flowering time and inflorescence architecture (Table 6).

Most of the genes harboring market-trait associations for the related traits in waterlogging treatment conditions are known to play a role in the regulation of waterlogging or other abiotic stress responses (Table 7). The genes HORVU2Hr1G072140, encoding Uridylate kinase, and HORVU2Hr1G013400, encoding Pseudo-response regulator 7 (PRR7), contain significant markers associated with BIO and are known to play a role in the response to abiotic stress, such as salinity, cold and oxidative stress (Table 7). The four genes HORVU6Hr1G070750 (annotated as E3 ubiquitin-protein ligase makorin), HORVU4Hr1G090640 (E3 ubiquitin-protein ligase RFWD3), HORVU4Hr1G000090 (RING/U-box superfamily protein), and HORVU2Hr1G094030 (Ubiquitin-conjugating enzyme 3) associated with SP, GP, and KWP regulate abiotic stress signaling pathways, such as in waterlogging or flooding conditions (Table 7). Also, the associated genes HORVU5Hr1G083110 (Leucine-rich repeat receptor-like kinase family protein) and HORVU2Hr1G111780 (Receptor-like protein kinase 4) are known to be involved in abiotic stress responses, including drought, salt, cold, toxic metals and other stresses. The gene HORVU2Hr1G025510 (B3 domain-containing protein), associated with SP, is involved in abiotic stress and disease resistance signaling pathways. The gene HORVU4Hr1G061070 (C2H2 zinc finger protein) associated with GP and KWP, participates in mechanisms of tolerance to salinity, osmotic, cold, drought, oxidative and high-light stress response (Table 7). The gene HORVU3Hr1G022270 (Pentatricopeptide repeat 336), associated with PH, is known to regulate plant responses to abiotic stresses (Table 7). The significant markers associated with WLS were located inside the genes encoding PRR7 and RING/U-box superfamily protein, and the genes HORVU4Hr1G069280 (Alpha-L-fucosidase 2), involved in the response to waterlogging, drought and salinity stresses, and HORVU1Hr1G017900 (Phytochrome-interacting factor 3), which regulates the plant response to drought and salt stresses (Table 7).

In the relative dataset, the significant markers JHI-Hv50k-2016-20766 and JHI-Hv50k-2016-21022 associated with SP, were inside the genes HORVU1Hr1G024060 (Arginine/serine-rich splicing factor 35) and HORVU1Hr1G036060 (tRNA pseudouridine synthase A1), respectively, that play important roles in development and response to abiotic stresses (Table 8). The role of the gene HORVU2Hr1G114940, encoding Cyclic nucleotide-gated channel 8, contains significant markers associated with KWP and is known to play a crucial role in pathways related to cellular ion homeostasis, development, and defense against biotic and abiotic stresses. The gene HORVU7Hr1G022410, encoding RNA-binding protein mde7, was associated with PH and has functional roles during growth, development, and abiotic stress responses in plants (Table 8). Additionally, the genes HORVU5Hr1G093390 and HORVU5Hr1G096320 were harboring markers associated with SP and were also identified in the control dataset harboring markers associated with PH, BIO, and KWP. The genes HORVU1Hr1G017900, HORVU4Hr1G000090, and HORVU4Hr1G069280 were harboring markers associated with WLS and also were identified in the waterlogging dataset associated with the same trait. Finally, the gene HORVU2Hr1G013400, encoding PRR7, contained markers associated with WLS in the waterlogging treatment and relative datasets, and BIO in the control dataset (Tables 6–8).

## Discussion

Waterlogging is becoming one of the challenging issues for modern agriculture globally. The development of tolerant cultivars with enhanced resilience to waterlogging stress has increasing importance to reduce the yield penalty. In this study, GWAS was performed based on linkage disequilibrium on a worldwide spring barley collection using control, waterlogging treatment and relative datasets for identifying QTL associated with yield-related traits and waterlogging tolerance.

### Diverse Phenotypic Variation and Waterlogging Tolerant Barley Genotypes

In the present study, the barley collection assembled showed significant phenotypic variation, as well as highly genotypic differences, for all traits after waterlogging stress treatment, including BIO, SP, GP, KWP, PH, and WLS, except CABC and CCC. These results suggest that there is a good potential that these genotypes can be used to mine alleles for waterlogging tolerance for introgression into breeding barley lines for waterlogging tolerance improvement. Waterlogging stress considerably reduced BIO, SP, GP, KWP, PH, CABC, and CCC for all genotypes in response to waterlogging stress as expected, and it is consistent with earlier studies (Li et al., 2008; Xue et al., 2010). Significant negative correlations were found between WLS and all other traits.

The barley genotype Deder2 from Ethiopia showed a tolerant response to waterlogging stress, while the response of the genotypes Yerong from Australia, TR 587 and CDC Select from Canada, Champion, Xena, and TR 987 from the USA, and Harumaki Rokkakumugi from North Corea, was more moderate. Some of these barley genotypes (e.g., Deder2, Harumaki Rokkakumugi, and Yerong) were previously reported (Takeda, 1989; Li et al., 2008) to be tolerant to waterlogging stress while the others, which are modern cultivars (Canadian Food Inspection Agency, 2021; Washington State Crop Improvement Association, 2021; Westland Seed, 2021) and elite breeding lines, were not reported before and might represent novel sources of tolerance.

### Genome-Wide Association Study Analysis

The GWAS is a powerful approach to locate common alleles associated with phenotypes with much higher resolution than linkage mapping because they reflect historical recombination events in broad-based diversity panels (Nordborg and Weigel, 2008). In this study, three statistical models were compared to assess their ability to map QTL and identify SNPs associated with waterlogging tolerance. Finally, we selected the MLM + Q + K approach, which accounts for both population structure (STRUCTURE analyses) and K matrix, because of its statistical power to control false-positives associations, which has been used successfully in barley (Pasam et al., 2012; Fan et al., 2016; Jabbari et al., 2018) and maize (Yu et al., 2006). Population structure and familial relatedness can result in false positives in GWAS. Therefore, when GWAS is conducted, these parameters need to be considered in the model. In the present study, the level of the genetic structure of the panel was assessed by the NJ tree, PCA, and STRUCTURE analyses and all showed that the investigated genotypes are structured into three principal groups. This provided additional confidence given that most of the barley population structure studies use only two of these methods, STRUCTURE and PCA, to confirm their results (Varshney et al., 2012; Long et al., 2013; Fan et al., 2016; Zhou et al., 2016; Bengtsson et al., 2017; Jabbari et al., 2018; Thabet et al., 2018; Milner et al., 2019; Mwando et al., 2020; Ye et al., 2020). Moreover, the LD decay value identified (1.46 Mbp at *r*^2^ = 0.1) suggested that the marker coverage is adequate for further GWAS analysis. A wide range of levels of LD decay, 2–10 cM, was reported by previous studies of worldwide barley collections (Comadran et al., 2009; Zhang et al., 2009; Pasam et al., 2012; Varshney et al., 2012; Long et al., 2013; Zhou et al., 2016). Comparison to any of these studies is hard to be made due to several factors such as size and diversity of the germplasm used, type and number of molecular markers, and measurement unit. Recently, Mwando et al. (2020) reported a LD decay of 3.5 Mbp (*r*^2^ = 0.2) in 350 barley accessions using 24,138 DArTseq and SNP markers. While this time the measurement unit is the same (Mbp) the results are not directly comparable to our study either, mainly due to the different germplasm assessed. Nevertheless, the work conducted by Mwando et al. (2020) demonstrated successful association mapping was achieved with a lower number of molecular markers (24,138 vs. 35,926) than used in our study.

The overall GWAS was able to identify significant QTL in all control, waterlogging treatment and relative datasets for six (BIO, SP, GP, KWP, PH, and WLS) out of the eight traits measured. No significant QTL were detected for CABC and CCC in the tested conditions. Chlorophyll is one of the major chloroplast components for photosynthesis, and relative chlorophyll content has a positive relationship with photosynthetic rate (Guo et al., 2008). An earlier study reported the identification of QTL for chlorophyll fluorescence in barley under low oxygen concentration in hydroponics to simulate waterlogging but not for chlorophyll content or chlorophyll (Bertholdsson et al., 2015).

### Identification of Known Waterlogging-Related QTL by GWAS

So far, several QTL mapping studies have been conducted using linkage mapping analysis in barley and many QTL associated with waterlogging tolerance have been successfully mapped using bi-parental linkage mapping based on various waterlogging related traits (Li et al., 2008; Xue et al., 2010; Zhou, 2011; Xu et al., 2012; Zhou et al., 2012; Bertholdsson et al., 2015; Broughton et al., 2015; Zhang et al., 2016; Gill et al., 2017, 2019; Zhang X. et al., 2017). These studies used DH populations from bi-parental crosses of contrasting phenotype parents for waterlogging. Direct comparisons of our GWAS findings with those studies are intricate, as the marker-trait linkages and chromosomal locations we identified were based on a worldwide barley collection not previously investigated for waterlogging traits. Moreover, different genotyping technologies and different linkage maps have been used in some of the previous studies, so the comparison is approximated. In general, our GWA mapping was highly consistent with those previous waterlogging tolerance QTL mapping studies conducted in bi-parental populations, and many QTL were identified for the same or related traits at similar positions, which confirmed the importance of the loci identified in the present study.

Some of the waterlogging-related QTLs detected in the waterlogging treatment dataset in our study are positioned closer to previously identified waterlogging stress-related QTLs for similar traits (Xue et al., 2010; Xu et al., 2012; Broughton et al., 2015; Ma et al., 2015). SP trait was associated with genomic regions related to the markers JHI-Hv50k-2016-3532 (at 3 Mbp on 1H), JHI-Hv50k-2016-109151 (at 662 Mbp on 2H) and JHI-Hv50k-2016-161633 (at 32 Mbp on 3H) were also associated with the related traits shoot fresh weight (QHSFW.1H) and tiller number (QHTiller.3H) in the Franklin x YYXT mapping population (Broughton et al., 2015), and grains per spike (GSw1.1 and GSw1.2) in Franklin x Yerong mapping population (Xue et al., 2010). The marker JHI-Hv50k-2016-3532 was also associated with the QTL for salinity and waterlogging tolerance (QSlww.YG.1H-1) in a DH population of Gairdner × YSM1 (Ma et al., 2015). The marker JHI-Hv50k-2016-109151 was also closely positioned near the QTL tfsur-1 which is associated with plant survival in the TX9425 × Franklin mapping population (Li et al., 2008). One of the genomic regions associated with KWP, related to the marker JHI-Hv50k-2016-127867 located at 724 Mbp on 2H was coincident with the previous identified QTL (SLw2.2) for spike length in the Franklin x Yerong population (Xue et al., 2010). Zhou (2011) also reported two QTL (QWL.YeFr.2H.2 and WL5.3) associated with waterlogging tolerance score, which is positioned near the marker JHI-Hv50k-2016-127867. WLS trait was associated with BOPA2_12_30872 located at 29 Mbp on 2H. This genomic region was previously detected in two different populations, TX9425 x Naso Nijo (Xu et al., 2012) and YSM1 x Gairdner (Ma et al., 2015), for the same trait. Additionally, in our study BIO was also associated with the same marker that was located on the genomic region 29.1–29.7 Mbp on chromosome 2H. Interestingly, in our study, this same region was also associated with the traits GP, KWP, and PH (JHI-Hv50k-2016-73570 and JHI-Hv50k-2016-73689).

Other waterlogging-related QTL detected in our study were identified in previous waterlogging stress studies but associated with different traits (Li et al., 2008; Xue et al., 2010; Zhou, 2011; Xu et al., 2012; Zhou et al., 2012; Broughton et al., 2015; Ma et al., 2015; Gill et al., 2017). For example, the traits SP, GP and KWP were associated with the genomic region 568.0–569.3 Mbp on 5H that was coincident for the QTL yfsur-2 for plant survival under waterlogging in the DH population of Yerong × Franklin (Li et al., 2008).

In our study, we identified five QTL in the relative dataset that were positioned closer to previously identified waterlogging stress-related QTLs for similar traits (Xu et al., 2012; Broughton et al., 2015; Ma et al., 2015). SP was associated with four QTL, QSP.1H-2, QSP.1H-3, QSP.1H-4, and QSP.1H-5, that were also associated with the related trait shoot dry weight (QHSDW.1H) in the Franklin x YYXT mapping population (Broughton et al., 2015). The QTL QWLS.4H-2 was associated with WLS and was also present in the waterlogging treatment dataset. Other waterlogging tolerance-related QTL detected in our study were identified in previous waterlogging stress studies but associated with different traits (Xue et al., 2010; Zhou, 2011; Xu et al., 2012; Broughton et al., 2015; Ma et al., 2015).

### Identification of Novel Waterlogging-Related QTL by GWAS

Among the 37 QTL detected under waterlogging treatment conditions, 13 QTL were detected on genomic regions where no waterlogging-related QTL have been previously reported in barley. These 13 QTL located in 10 different genomic regions, probably represents novel loci for waterlogging stress. Two significant associated markers, JHI-Hv50k-2016-68186 and JHI-Hv50k-2016-68266, were identified on 2H at 16 Mbp. The first marker was associated with the trait KWP and the second with SP. On chromosome 4H at 0.37, 512, 569, and 645 Mbp, four markers, JHI-Hv50k-2016-225852, JHI-Hv50k-2016-249670, BOPA1_3549-743, and JHI-Hv50k-2016-276624, were identified. The first marker was associated with SP and WLS, the second marker with GP and KWP, the third marker with WLS and the last marker with SP. On chromosome 6H at 492, 554, and 562 Mbp, three markers, JHI-Hv50k-2016-410329, JHI-Hv50k-2016-421359, and JHI-Hv50k-2016-424341, were associated with GP, WLS, and KWP, respectively. The marker JHI-Hv50k-2016-449124 was associated with GP and KWP on 7H at 13 Mbp.

In the relative dataset, six QTL (QPH.2H-1, QSP.1H-1, QSP.5H-1, QWLS.4H-1, QWLS.4H-2, and QWLS.6H) out of 20 were detected on genomic regions that have not been reported in previous waterlogging-related QTL studies on barley conducted using bi-parental populations and they probably represent novel loci for waterlogging tolerance. SP was associated with the markers JHI-Hv50k-2016-20766 and JHI-Hv50k-2016-312394, located on chromosome 1H at 107 Mbp and 5H at 532 Mbp, respectively. The marker BOPA2_12_30631 was associated with PH on 2H at 18 Mbp. For WLS, three markers were found to be associated, JHI-Hv50k-2016-225850 and BOPA1_3549-743, located on 4H at 0.37 and 569 Mbp, respectively, and JHI-Hv50k-2016-421359 on 6H at 554 Mbp. The genomic regions at 0.37 and 569 Mbp on 4H and 554 Mbp on 6H were co-localized in waterlogging treatment and relative datasets, associated with WLS. Interestingly, QWLS.4H-2 is positioned relatively close to the QTL for aerenchyma formation (QTL-aerenchyma) and root porosity (QTL-rp4H) (Zhang et al., 2016).

### Waterlogging-Related Candidate Genes

In the present study, 92 markers significantly associated with yield-related traits were identified in control conditions, which were located along 28 QTL regions on chromosomes 2H, 3H, 5H, 6H, and 7H; 63 significant markers were identified under waterlogging treatment conditions and mapped along 24 QTL regions on all chromosomes in the barley genome; while 51 significant markers located in 17 QTL regions distributed along chromosomes 1H, 2H, 4H, 5H, 6H, and 7H were identified in the relative data set. Among those QTL, we detected possible candidate genes that were associated with the measured traits under the different growing conditions, i.e., control, waterlogging treatment, and the relative difference between these two conditions.

Genes affected by waterlogging stress and involved in the tolerance of barley to this stress are most valuable in waterlogging breeding programs to develop and improve the efficiency of waterlogging-tolerant barley varieties. In our study, most of the potential candidate genes containing significant markers under waterlogging treatment conditions were detected on 2H and 4H associated with BIO, GP and PH. However, for the relative dataset, chromosome 1H contained most of the potential candidate genes, followed by 2H, 4H, and 5H. Four QTL that appears to harbor genes associated with abiotic stress tolerance were detected on both waterlogging treatment and relative datasets to be associated with WLS. The most significant two are QWLS.2H, harboring the gene PRR7 (HORVU2Hr1G013400) on 2H at 29.1 Mbp, is potentially similar to the reported QTL for membrane potential QMP.TxNn.2H (Gill et al., 2017); and the novel QWLS.4H-2, harboring the gene Alpha-L-fucosidase 2 (HORVU4Hr1G069280) on 4H at 569.7 Mbp, that is closely located to the reported QTL for aerenchyma formation (Zhang et al., 2016). PRR7 has a central role in the abiotic stress response and influences the regulation of flowering time and ABA-related processes, including control of genes affecting salinity, cold and oxidative stress response (Liu et al., 2013). This gene harbored the BOPA2_12_30872 marker that was also associated with BIO under waterlogging stress conditions. Alpha-L-fucosidase 2 is known to be involved in the breakdown of cell wall polymers and was previously reported to be upregulated in tolerant genotypes of maize, sesame, and chickpea in response to waterlogging, drought and salinity stresses, respectively (Thirunavukkarasu et al., 2013; Dossa et al., 2017; Kaashyap et al., 2018). These results indicated the reliability of the QTL in this study. The other two genes were detected on 1H and 4H. The Transcription factor PIF3 on QWLS.1H regulates the plant response to drought and salt stresses in maize (Gao et al., 2015) and plays a positive role in submergence-induced hypocotyl elongation in Arabidopsis (Wang et al., 2020). RING/U-box superfamily protein on the novel QWLS.4H-1 is involved in the ubiquitination reaction, a crucial mechanism that regulates signal transduction in diverse biological processes, including abiotic stress signaling pathways, such as in waterlogging or flooding conditions (Voesenek and Bailey-Serres, 2015; Loreti et al., 2016). This strong ubiquitin response is a robust indicator of changing physiological situation, by repurposing proteins through proteolysis. Additionally, the novel QWLS.6H detected only in waterlogging stress conditions harbored Receptor kinase 2 that belongs to the largest group within the receptor-like kinase (RLK) superfamily in plants and had been reported as having a main role in developmental processes, signaling networks and disease resistance. Many RLKs are involved in abiotic stress responses, including drought, salt, cold, toxic metals and other stresses (reviewed in Ye et al., 2017). For example, a hypersensitive response was observed in response to salt and heat stress in Arabidopsis (Park et al., 2014). The homolog of the gene HORVU5Hr1G071230, harboring QSP.5H-1 on 5H at 532 Mbp, in Arabidopsis it is characterized as a cell wall integrity/stress response component.

Additionally, in our previous study, an RNA-Sequencing analysis was conducted to explore the mechanisms involved in the responses of two barley genotypes with tolerant, Deder2, and moderately-tolerant, Yerong, responses to waterlogging stress (Borrego-Benjumea et al., 2020). One of the top highly expressed differentially expressed genes (logFC ≥ ±4 and adjusted *P* < 0.05) in the roots of waterlogged Deder2 and Yerong, was the upregulated gene Trichome birefringence-like 19 (8.47 logFC) which is very close to the marker JHI-Hv50k-2016-276624. This marker in the current study is associated with SP in the waterlogging treatment conditions. The underlying function of this gene is the ubiquitous modification of cell wall polymers by acetylation and is known to play a structural role in plant growth and microorganism and environmental stresses defenses (Nafisi et al., 2015), such as salinity and cold (Anantharaman and Aravind, 2010). The marker JHI-Hv50k-2016-249670, associated with GP and KWP in the waterlogging treatment conditions, is in the surroundings of the upregulated gene encoding the protein very-long-chain3-oxoacyl-CoA reductase 1 (5.26 logFC). This protein is required for the elongation of fatty acids precursors of sphingolipids, triacylglycerols, cuticular waxes and suberin, and play a role in the stress adaptation in rice. The downregulated gene Copalyl diphosphate synthase 2 (−7.34 logFC) is located very close to the marker JHI-Hv50k-2016-322288 associated with KWP in the waterlogging treatment conditions. This gene responds to arsenic detoxification in rice and it is involved in the plant adaptive responses to arsenic stress (Singh et al., 2017). The marker JHI-Hv50k-2016-3532, associated with SP in the waterlogging treatment conditions, is positioned in the surroundings of the downregulated gene encoding the protein Dirigent protein 21 (−4.76 logFC). This protein is involved in the defense response against salt and drought stress of pepper (Khan et al., 2018).

Further analysis is necessary to validate the associated candidate genes. However, this study represents the starting point of the discovery of candidate genes associated with waterlogging tolerance as well as the development of useful gene-based functional markers for barley breeding to speed up the development of waterlogging tolerant barley cultivars.

## Conclusion

GWAS based on high-density SNP markers represents a powerful approach for dissecting complex quantitative traits. In this study, 247 worldwide spring barley genotypes were evaluated for yield components-related traits under control and waterlogging treatment conditions in the field, as well as the relative difference between these two conditions, and were genotyped using Barley 50K iSelect SNP Array. GWAS analysis showed that a total of 92, 63, and 51 markers were significantly associated with BIO, SP, GP, KWP, PH, and WLS traits in the control, waterlogging treatment, and relative datasets, respectively. Seventeen significant associations and eight potential candidate genes were detected for the relative dataset. Also, six novel QTL (QPH.2H-1, QSP.1H-1, QSP.5H-1, QWLS.4H-1, QWLS.4H-2, and QWLS.6H) were detected on genomic regions that have not been reported in previous waterlogging-related QTL studies on barley and they probably represent novel loci for waterlogging tolerance. These findings provide useful information for waterlogging tolerance in barley by marker-assisted selection in the future. For further research, it will be necessary the validation of the associated candidate genes and the development of markers based on associated SNPs.

## Data Availability Statement

The original contributions presented in the study are included in the article/Supplementary Material, further inquiries can be directed to the corresponding author/s.

## Author Contributions

AB-B, AB, and MZhu: conceptualization. AB-B, AB, AC, JT, MZhu, and MZho: methodology and writing—review and editing. AB-B, AC, and JT: software. AB-B and AB: writing—original draft preparation. AB: supervision, project administration, and funding acquisition. All authors have read and agreed to the published version of the manuscript.

## Conflict of Interest

The authors declare that the research was conducted in the absence of any commercial or financial relationships that could be construed as a potential conflict of interest.

## Publisher's Note

All claims expressed in this article are solely those of the authors and do not necessarily represent those of their affiliated organizations, or those of the publisher, the editors and the reviewers. Any product that may be evaluated in this article, or claim that may be made by its manufacturer, is not guaranteed or endorsed by the publisher.

## Abbreviations

BIO: above-ground dry Biomass
CABC: chlorophyll a+b content
CCC: chlorophyll carotenoids content
GP: number of grains per plant
GWAS: Genome-wide association study
KWP: kernel weight per plant
LD: linkage disequilibrium
PH: plant height
QTL: quantitative trait loci/locus
SNP: single nucleotide polymorphism
SP: number of spikes per plant
WLS: waterlogging score.
