# Supplementary material for: Genome-Wide Association Study of Waterlogging Tolerance in Barley (Hordeum vulgare L.) Under Controlled Field Conditions
Source: Front Plant Sci. 2021 Aug 26;12:711654. doi: 10.3389/fpls.2021.711654 (PMC8427447; doi:10.3389/fpls.2021.711654)
Supplement: Supplementary file 3 [file Data_Sheet_3.docx]

Supplementary Material

**Figures**


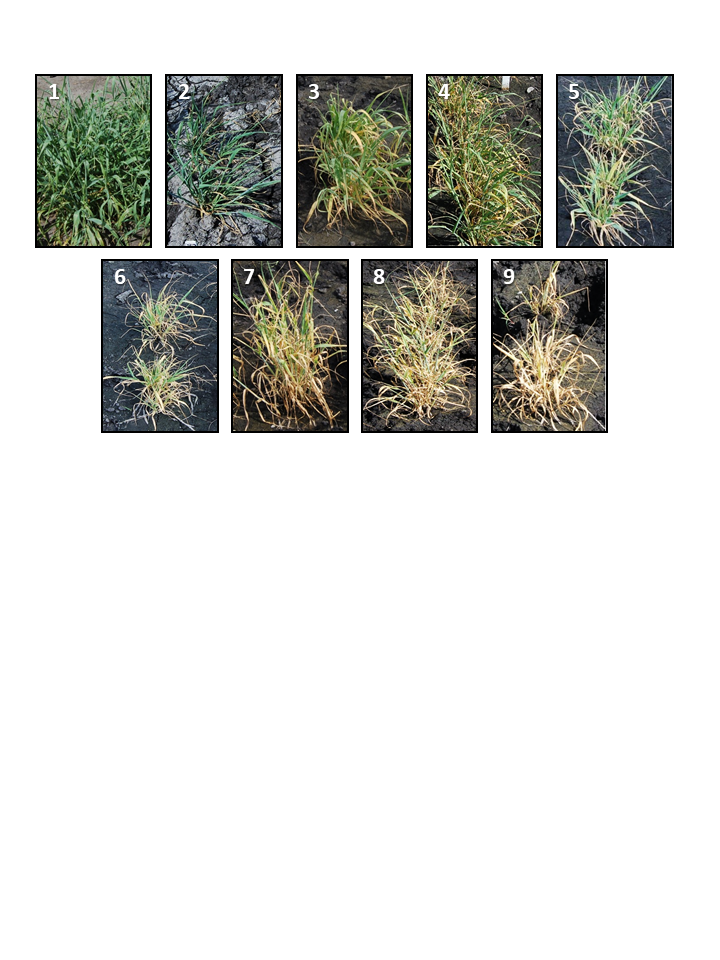


**Supplementary Figure S1.** Waterlogging score scale (1-9), determined based on plant survival and leaf chlorosis (1 = not affected by waterlogging, 9 = plants died from waterlogging).


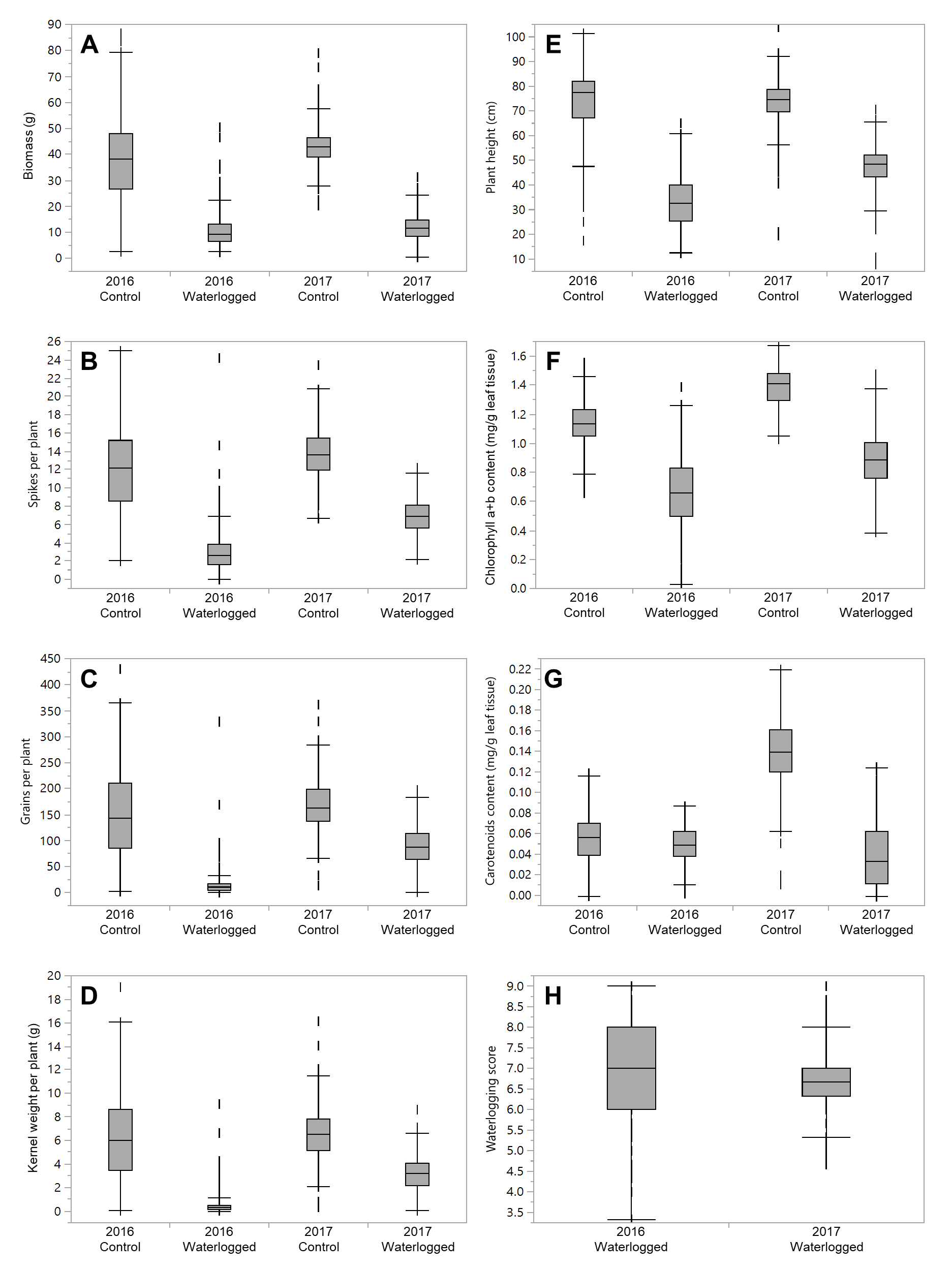


**Supplementary Figure S2.** Boxplots of Biomass = BIO (A), Spikes per plant = SP (B), Grains per plant = GP (C), Kernel weight per plant = KP (D), Plant height = PH (E), Chlorophyll a+b content = CABC (F), Carotenoids content = CCC (G), and Waterlogging score = WLS (H) of the spring barley collection evaluated in the control and waterlogging treatment under field conditions.


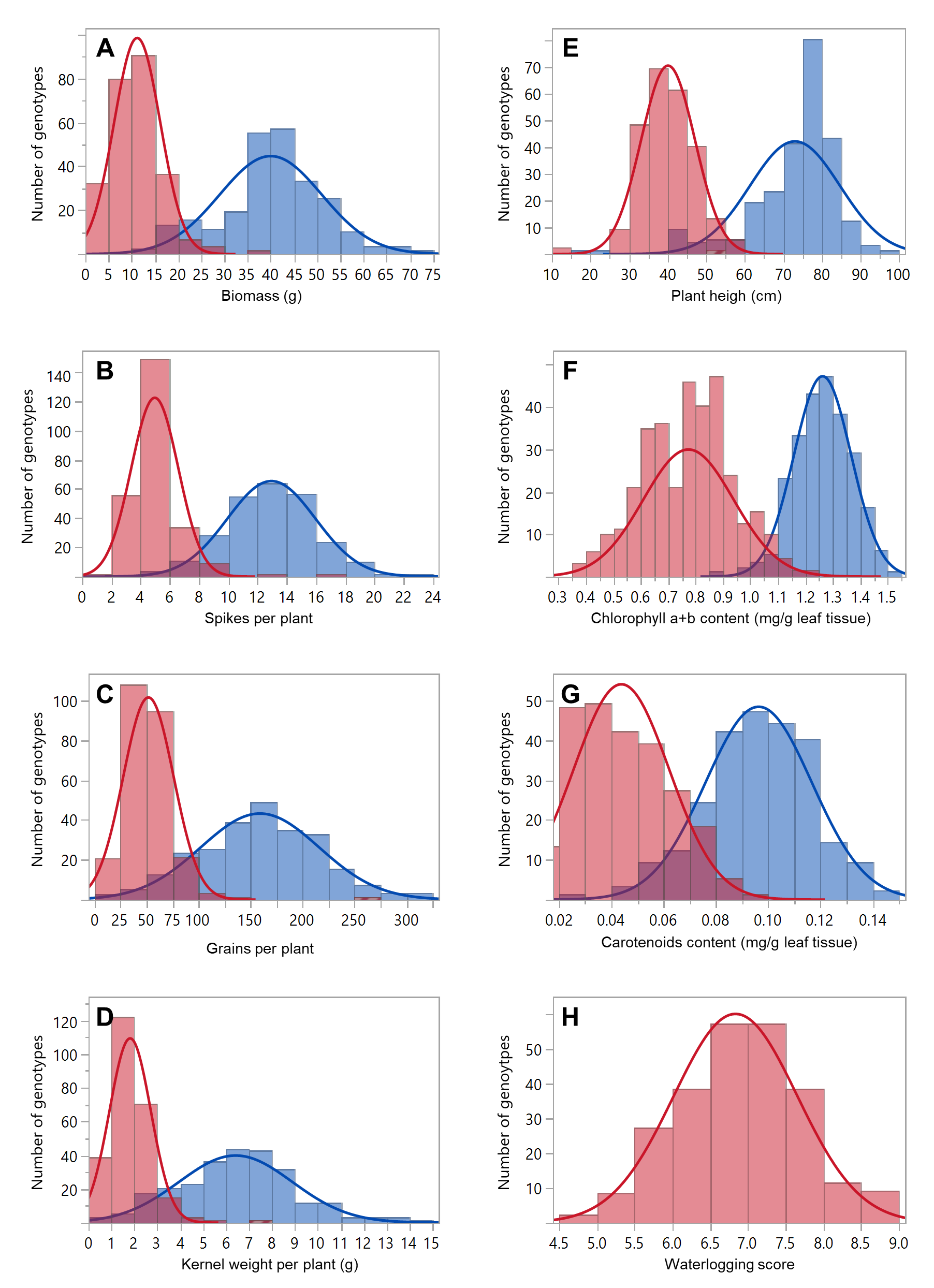


**Supplementary Figure S3.** Frequency distribution of phenotypic variation of Biomass = BIO (A), Spikes per plant = SP (B), Grains per plant = GP (C), Kernel weight per plant = KP (D), Plant height = PH (E), Chlorophyll a+b content = CABC (F), Carotenoids content = CCC (G), and Waterlogging score = WLS (H) of the spring barley collection evaluated in the control (blue) and waterlogging treatment (red) under field conditions.


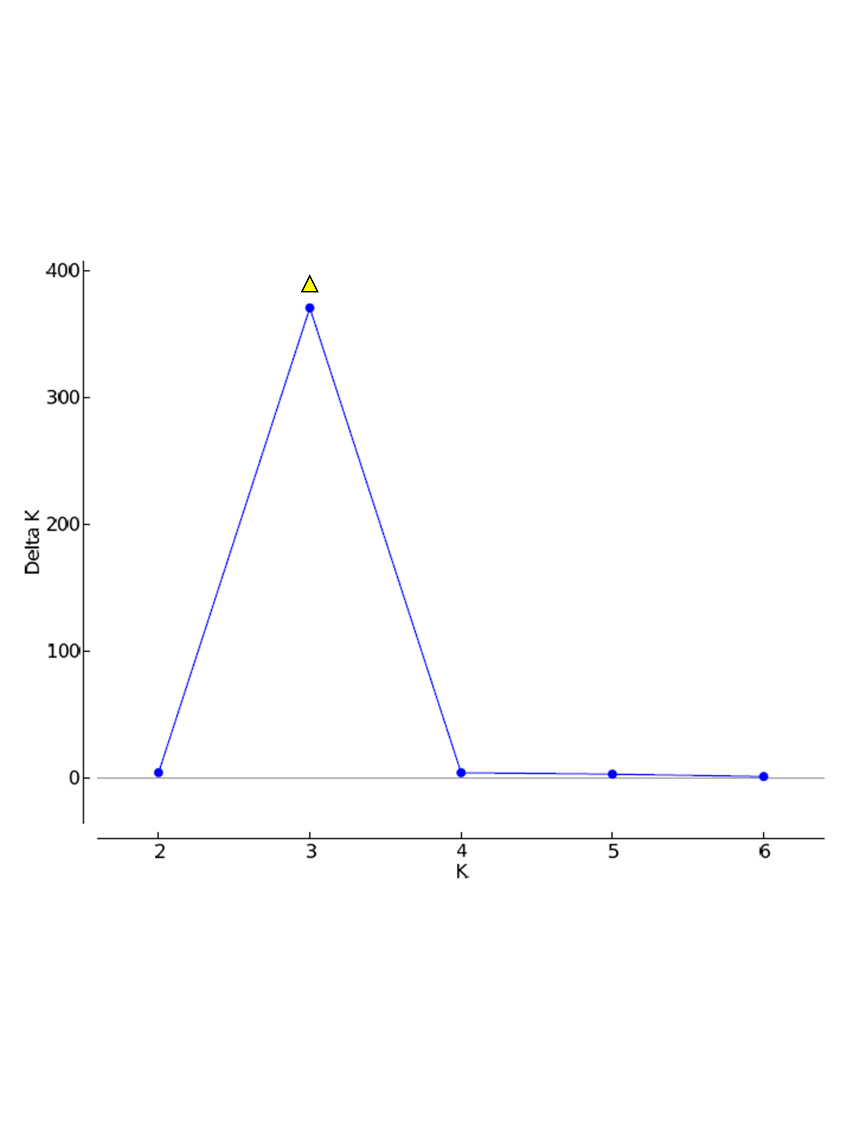


**Supplementary Figure S4.** Estimation of the ideal number of subpopulations clusters (*∆K*) in the population of 247 spring barley genotypes. The cluster parameter *K* was set from 1 to 7. The yellow triangle at *K*=3 indicates the correct number of clusters in this dataset.


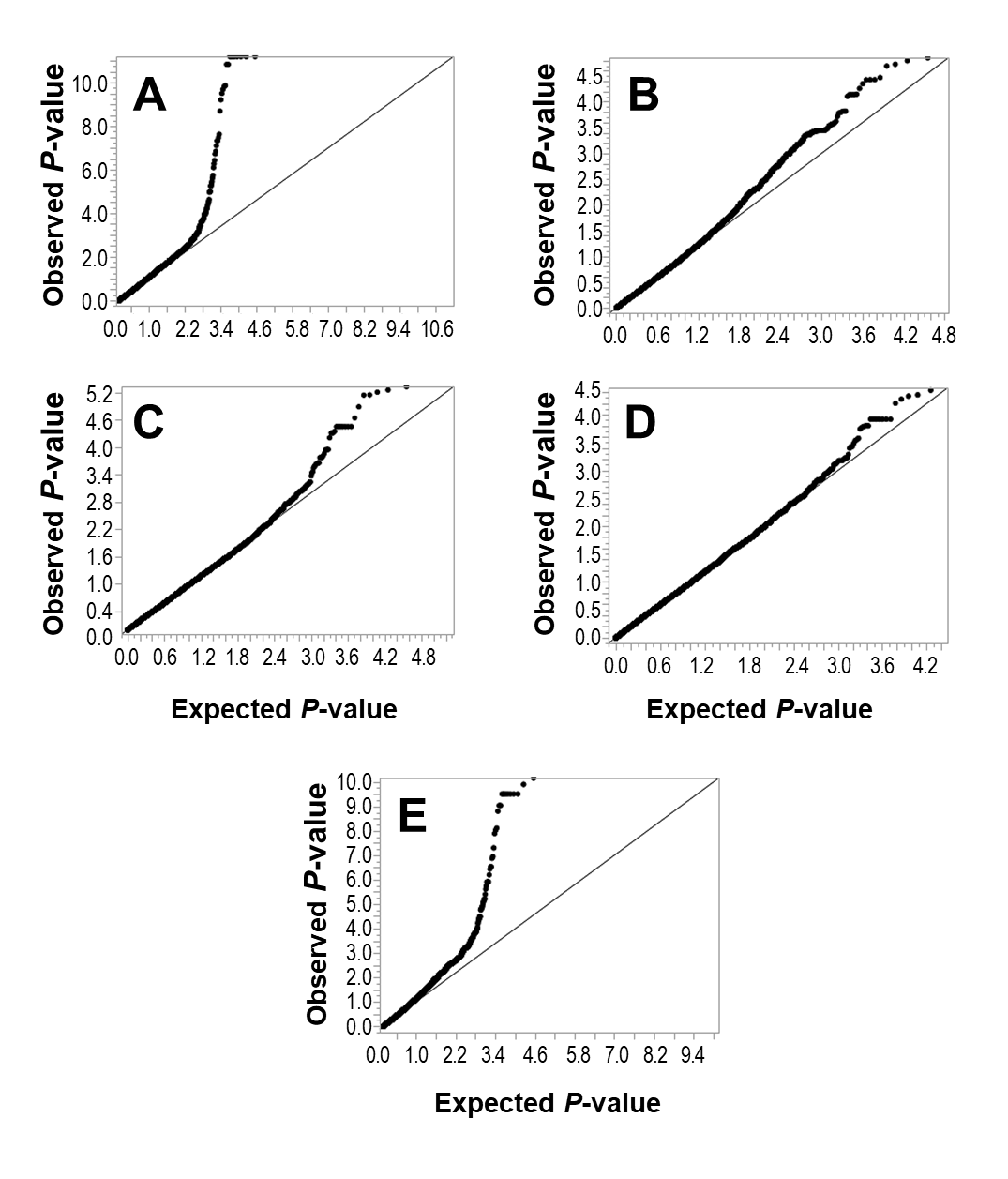


**Supplementary Figure S5.** Quantile-quantile (QQ) plots resulting from the SNP-based GWAS in overall control under field conditions for Biomass (BIO), Spikes per plant (SP), Grains per plant (GP), Kernel weight per plant (KWP), and Plant height (PH) are shown in A, B, C, D, and E, respectively. GWAS was performed using the MLM (Q + K) model in JMP Genomics for the field traits.


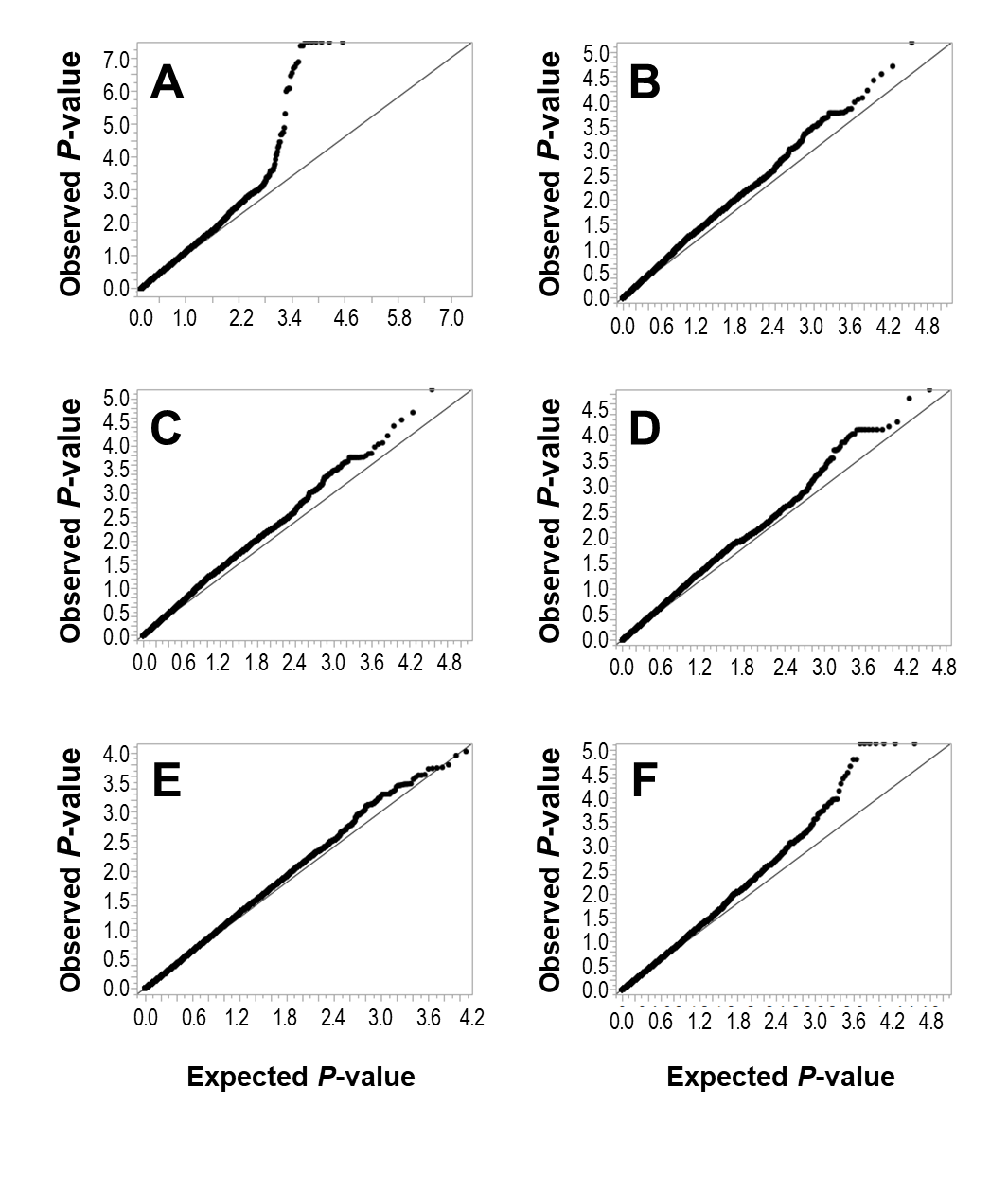


**Supplementary Figure S6.** Quantile-quantile (QQ) plots resulting from the SNP-based GWAS in waterlogging treatment under field conditions for Biomass (BIO), Spikes per plant (SP), Grains per plant (GP), Kernel weight per plant (KWP), Plant height (PH), and Waterlogging score (WLS) are shown in A, B, C, D, E, and F, respectively. GWAS was performed using the MLM (Q + K) model in JMP Genomics for the field traits.


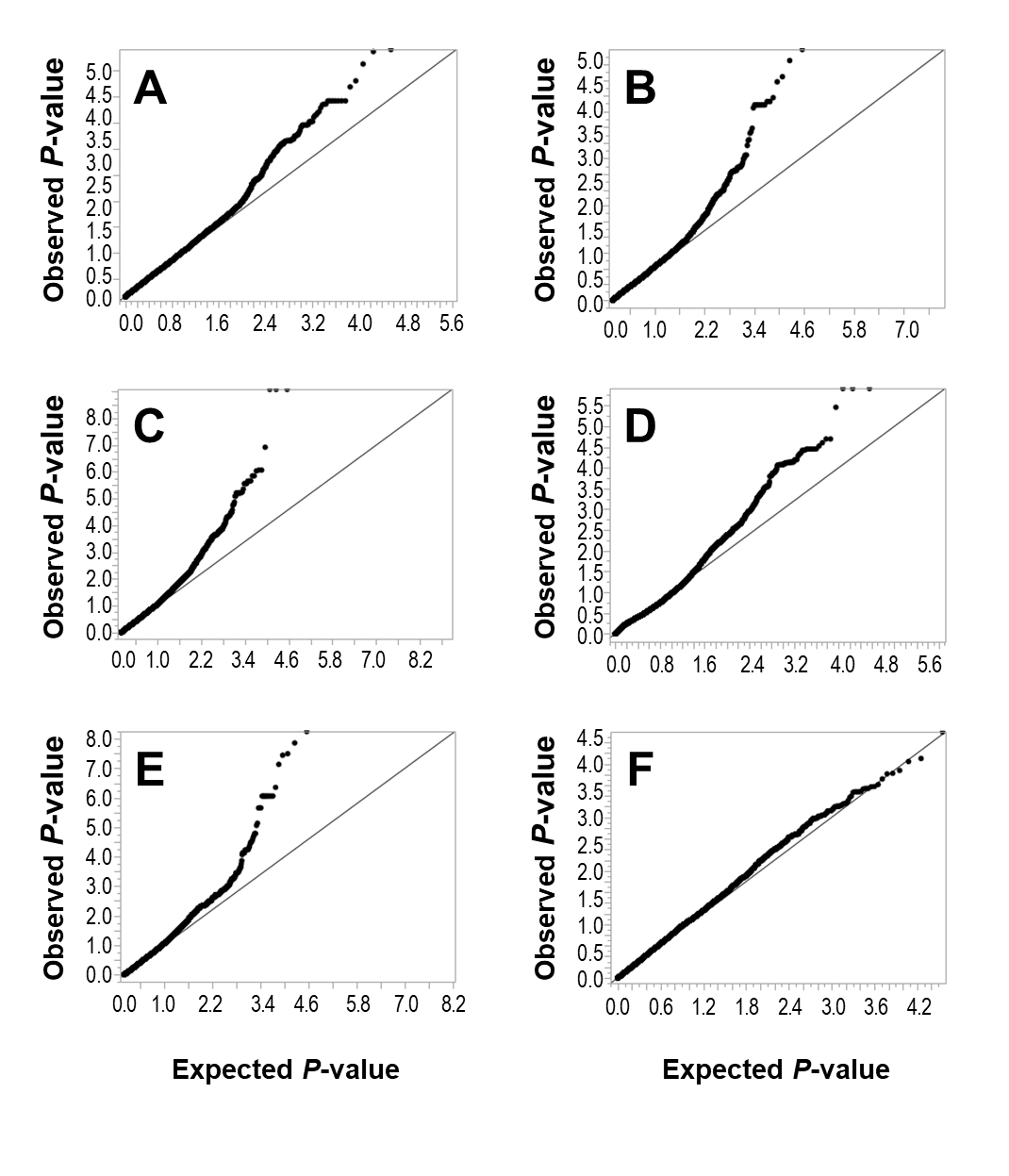


**Supplementary Figure S7.** Quantile-quantile (QQ) plots resulting from the SNP-based GWAS identified in the relative dataset for Biomass (BIO), Spikes per plant (SP), Grains per plant (GP), Kernel weight per plant (KWP), Plant height (PH), and Waterlogging score (WLS) are shown in A, B, C, D, E, and F, respectively. GWAS was performed using the MLM (Q + K) model in JMP Genomics for the field traits.

**Supplementary Figure S8.** Location of QTL regions identified in the relative dataset on chromosomes on a barley physical map. BIO indicates Biomass, SP indicates Spikes per plant, GP indicates Grains per plant, KWP indicates kernel weight per plant, PH indicates Plant height, WLS indicates Waterlogging score.
